# Analysis of Epigenetic Age Predictors in Pain-Related Conditions

**DOI:** 10.3389/fpubh.2020.00172

**Published:** 2020-06-09

**Authors:** Katarzyna Malgorzata Kwiatkowska, Maria Giulia Bacalini, Claudia Sala, Helena Kaziyama, Daniel Ciampi de Andrade, Rossana Terlizzi, Giulia Giannini, Sabina Cevoli, Giulia Pierangeli, Pietro Cortelli, Paolo Garagnani, Chiara Pirazzini

**Affiliations:** ^1^Department of Experimental, Diagnostic and Specialty Medicine, University of Bologna, Bologna, Italy; ^2^IRCCS Istituto delle Scienze Neurologiche di Bologna, Bologna, Italy; ^3^Department of Physics and Astronomy, University of Bologna, Bologna, Italy; ^4^Department of Neurology, Pain Center, LIM 62, University of São Paulo, São Paulo, Brazil; ^5^Pain Center, Instituto do Câncer do Estado de São Paulo, São Paulo, Brazil; ^6^U. O. Neurologia, Ospedale Bufalini, Cesena, Italy; ^7^Department of Biomedical and Neuromotor Sciences, University of Bologna, Bologna, Italy; ^8^Department of Laboratory Medicine, Clinical Chemistry, Karolinska Institutet, Karolinska University Hospital, Stockholm, Sweden; ^9^Applied Biomedical Research Center (CRBA), Policlinico S.Orsola-Malpighi Polyclinic, Bologna, Italy; ^10^Unit of Bologna, CNR Institute of Molecular Genetics Luigi Luca Cavalli-Sforza, Bologna, Italy

**Keywords:** epigenetic aging, aging biomarker, epigenetic clock, chronic pain, pain sensitivity, fibromyalgia, headache, DNA methylation

## Abstract

Chronic pain prevalence is high worldwide and increases at older ages. Signs of premature aging have been associated with chronic pain, but few studies have investigated aging biomarkers in pain-related conditions. A set of DNA methylation (DNAm)-based estimates of age, called “epigenetic clocks,” has been proposed as biological measures of age-related adverse processes, morbidity, and mortality. The aim of this study is to assess if different pain-related phenotypes show alterations in DNAm age. In our analysis, we considered three cohorts for which whole-blood DNAm data were available: heat pain sensitivity (HPS), including 20 monozygotic twin pairs discordant for heat pain temperature threshold; fibromyalgia (FM), including 24 cases and 20 controls; and headache, including 22 chronic migraine and medication overuse headache patients (MOH), 18 episodic migraineurs (EM), and 13 healthy subjects. We used the Horvath's epigenetic age calculator to obtain DNAm-based estimates of epigenetic age, telomere length, levels of 7 proteins in plasma, number of smoked packs of cigarettes per year, and blood cell counts. We did not find differences in epigenetic age acceleration, calculated using five different epigenetic clocks, between subjects discordant for pain-related phenotypes. Twins with high HPS had increased CD8+ T cell counts (nominal *p* = 0.028). HPS thresholds were negatively associated with estimated levels of GDF15 (nominal *p* = 0.008). FM patients showed decreased naive CD4+ T cell counts compared with controls (nominal *p* = 0.015). The severity of FM manifestations expressed through various evaluation tests was associated with decreased levels of leptin, shorter length of telomeres, and reduced CD8+ T and natural killer cell counts (nominal *p* < 0.05), while the duration of painful symptoms was positively associated with telomere length (nominal *p* = 0.034). No differences in DNAm-based estimates were detected for MOH or EM compared with controls. In summary, our study suggests that HPS, FM, and MOH/EM do not show signs of epigenetic age acceleration in whole blood, while HPS and FM are associated with DNAm-based estimates of immunological parameters, plasma proteins, and telomere length. Future studies should extend these observations in larger cohorts.

## Introduction

Chronic pain is defined as a “pain which has persisted beyond normal tissue healing time” (1), a process that, in the absence of additional unfavorable factors, is expected to not exceed a period of 3 months. Chronic pain is common in both developed and developing countries (2, 3). In 2006, a large computer-assisted telephone survey reported that in European countries, the prevalence of chronic pain varied from 12 to 30%, with Spain, Ireland, and UK among the countries with the lowest prevalence, and Italy, Poland, and Norway among those with the highest prevalence (4). These country-dependent differences are probably triggered by multiple factors, including differences in pain perception and treatment, lifestyle, and age of the participants.

Accordingly, the etiology of chronic pain is multifactorial and embraces a broad range of factors that can be grouped in demographic, clinical, psychological, and lifestyle domains. Risk factors for chronic pain may not only trigger the onset of a persistent syndrome, but may also influence its eventual manifestation, having impact on different chronic pain dimensions like duration, localization, intensity, interference in daily life activities, or influence on emotional state. Advanced chronological age, female biological sex, feminine gender identity, deprived socio-economic status, unemployment, and adverse and unsatisfactory occupational situation are among the demographic positive risk factors for chronic pain (5–8). Although the reported prevalence rates tend to be higher in developing countries, the correlation between ethnicity and chronic pain is complex and the driving mechanisms are not clearly determined yet (9). In addition, cultural heritage and tradition with its practices and rituals are additional risk agents that modulate the attitudes toward the painful experience influencing the manifestation and/or perception of chronic condition (10). Among the clinical risk factors, the most pronounced one is the coexistence of another acute or chronic pain (11). Co-morbid physical and mental disorders, surgical, and medical interventions that have been undergone, increased BMI, and sleep disorders are the risk agents favoring persistent painful phenotypes (12–15). Also, several DNA variants that may be responsible for the genetic pre-disposition to develop pain have been identified (16). More than 150 genes have been already associated with pain-related conditions, among which are *COMT, OPRM, SNC9A, IL6*, or *TNFA*. The personal attitude and beliefs, concerns, and fears stimulate the development of the chronic pain conditions and can restrain or totally impede the recovery, as in the case of fear-avoidance model behavior in musculoskeletal pain disorders (17). Finally, the risk factors connected to lifestyle are smoking, alcohol use disorders, limited physical activity, and painogenic modern urban environment with, for example, low sun exposure or high air pollution (18–21). Additionally, the individual alimentary habits plausibly contribute to development and prevention of long-lasting pain disorders but the mechanism remains unclear (22).

As mentioned above, advanced age is a risk factor for chronic pain and often phenotypes of pre-mature aging are observed in patients. These manifestations of accelerated aging involve not only structural changes in the brain, like a total and regional decrease of gray matter (23–25), but also more systemic changes like a decrease in peripheral blood leukocyte telomere length (26) and increased inflammation (27–29).

Advances in recent research have led to the identification of a limited set of biomarkers that are considered potential biological age predictors (30), i.e., that are informative of the discrepancy between chronological age and biological age in conditions associated with successful (biological age deceleration) or unsuccessful (biological age acceleration) conditions. Potential markers of biological age include the analysis of telomere length, a brain age predictor based on structural neuroimaging [T1-weighted magnetic resonance imaging (MRI)], and different types of epigenetic clocks based on the DNA methylation (DNAm) values of specific CpG sites. In particular, epigenetic clocks have been extensively analyzed in physiological and pathological conditions (31) and an increase in predicted epigenetic age compared with chronological age has been associated to multiple conditions including neurological diseases (32, 33), progeroid syndromes (34–36) and, although in a less straightforward way, morbidity, and mortality (37, 38). Epigenetic clock measurements in whole blood have been associated with socio-cultural aspects, including education, lifestyle, and socio-economic status (38–41) and with exposure to stress and trauma (42, 43).

The “first generation” epigenetic clocks were developed on the basis of the association between DNAm and chronological age. The most used predictors were built using different training sets, which included large datasets of multiple tissues (44), whole blood (45), or human cell types used in *ex vivo* studies (35). Recently, more sophisticated epigenetic clocks have been built using not only chronological age but also clinical biomarkers that are informative of the quality of aging or associated with mortality more than age itself. The PhenoAge clock includes 10 variables (albumin, creatinine, serum glucose, C-reactive protein, lymphocyte percent, mean cell volume, red cell distribution width, alkaline phosphatase, white blood cell count, and age) (38), while the GrimAge is a composite biomarker based on the DNAm surrogates of seven plasma proteins and of smoking pack-years (40). Both PhenoAge and GrimAge outperformed previous epigenetic clocks in their associations with age-related conditions and mortality.

To the best of our knowledge, only one study investigated epigenetic age acceleration in chronic pain (46). The authors analyzed 20 individuals with chronic pain between 60 and 83 years and 9 age-matched controls and evaluated biological age acceleration by calculating the difference between Horvath's DNAm age and chronological age. A younger epigenome was observed in subjects that did not experience chronic pain in the past 3 months. Individuals characterized as emotionally stable, conscientious, and extrovert demonstrated lower epigenetic age. Epigenetic age acceleration was shown to be positively associated with higher experimental pain sensitivity and negatively associated with fluid cognition and memory, globally supporting an association between epigenetic age and chronic pain.

The aim of the present work is to further explore the association between epigenetic age and chronic pain, by investigating first- and second-generation epigenetic clocks and DNAm surrogates of plasma proteins, blood cell counts, and telomere length in different pain-related conditions for which methylation data are available.

## Materials and Methods

### Datasets

Our work involves DNAm data from three epigenome-wide studies investigating methylation patterns in pain-related phenotypes: heat pain sensitivity (HPS), fibromyalgia (FM), and headache syndromes comprising medication-overuse headache and episodic migraine. The characteristics of the datasets are provided in Table S1 and are summarized in the following paragraphs.

#### Heat Pain Sensitivity

The HPS dataset was acquired through Gene Expression Omnibus (GEO) NCBI repository (http://www.ncbi.nlm.nih.gov/geo/) under accession number GSE53128 (47). It includes DNAm data generated using the Infinium Human Methylation 450K BeadChip on whole blood from female monozygotic twins discordant for HPS, belonging to the British TwinsUK collection (48). Methylation data were available for 43 whole-blood samples. Three subjects were not considered in the analysis due to missing data and unfeasibility to assign them unequivocally to one of the phenotypic classes, thus leaving 20 twin pairs. The individuals ranged in age between 47 and 76 years old. The heat pain suprathreshold (HPST) scores were obtained with quantitative sensory testing (QST) and discordance was defined as a minimum difference of 2°C within the twin pairs. On the basis of HPST values, we assigned each participant to one of two phenotypic groups: high pain sensitivity (H), i.e., siblings with lower HPST values compared with their co-twin; low pain sensitivity (L), i.e., siblings with higher HPST values compared with their co-twin. The analysis of the HPS dataset was performed considering the entire cohort or dividing it into two subsets, including subjects younger than 60 years old (8 twin pairs) or older than 60 years old (12 twin pairs).

#### Fimbromyalgia

The FM dataset was retrieved from GEO NCBI repository under accession number GSE85506 (49). This pilot study assessed whole-blood DNAm in female patients with FM using the Infinium Human Methylation 450K BeadChip. It includes 24 cases and 23 age- and sex-matched controls recruited from the Brazilian population. The age range of the cohort was 19–80 years old. One healthy subject was not included in the analysis due to missing information on chronological age. Patients were classified as cases after neurological and psychiatric evaluation, verifying differential diagnosis according to current gold standard guidelines. In addition, FM-positive individuals were clinically characterized with a battery of tests and questionnaires: McGill Pain Questionnaire assessing sensory, affective, evaluative dimension of pain (MPQ_sensory, MPQ_affective, and MPQ_evaluative); Visual Analog Scale (VAS) reflecting the pain intensity; Brief Pain Inventory (BPI) evaluating the interference of painful experience with daily activities (total score) and registering the dosage and efficacy of pharmaceutical treatment (7th item of BPI questionnaire); FM Impact Questionnaire (FIQ) examining the impact of pain on different health domains; Pain Catastrophizing Scale (PCS) measuring a tendency to exaggerated negative attitudes in response to noxious stimuli. Three cases had missing values for the duration time of painful symptoms.

#### Headache

The Headache dataset is part of an exploratory GWAS longitudinal study on Italian subjects with painful cephalic phenotypes (50). According to the criteria defined by the International Headache Society 3rd edition (beta version), during the clinical examination, all participants were assigned to one of the following phenotypic groups: (i) chronic migraine and medication overuse headache patients (MOH), (ii) episodic migraine patients (EM), and (iii) healthy controls (HC). In this work, we focused on DNAm data collected at baseline time point (T0), which included 22 MOH (20 females, 2 males), 18 EM (17 females, 1 male), and 13 HC (8 females, 5 males). The age range of the subjects was between 24 and 69 years old. Whole-blood DNAm patterns were assessed by the Illumina Infinium Human Methylation EPIC BeadChip.

### Data Pre-processing

Raw data files (*.idat* format) from the three studies were downloaded and separately pre-processed using *minfi* package within Rstudio software (version 3.5.1) in Linux environment. *minfi* package provides tools for the analysis of Infinium DNA Methylation microarrays and can handle both 450k and EPIC arrays (51, 52). The pre-processing, quality control, and normalization steps were implemented as recommended by Maksimovic et al. (53). Probes with a detection *p*-value higher than 0.05 were recognized as failed. Only samples with at least 95% of successfully assessed probes were retained and probes that did not reach significant detection *p*-values in at least 99% of samples were filtered out. According to these filtering criteria, all the samples from the three cohorts were retained, while 3,493, 2,034, and 4,773 probes were removed in HPS, FM, and MOH/EM datasets, respectively.

### Calculation of DNAm Estimates

DNAm estimates were calculated using the New DNA Methylation Age Calculator, an open access tool available at https://dnamage.genetics.ucla.edu/ (44). Pre-processed methylation data were first normalized by the *preprocessQuantile* function implemented in minfi R package, as suggested in the Horvath's tutorial. Then, beta values matrixes were uploaded in the online tool, selecting the options “Advanced Analysis” and “Normalize Data,” as recommended in the software tutorial. Horvath's epigenetic age calculator returned as output a set of variables including different measures of biological age in blood and of epigenetic age acceleration in blood, DNAm-based surrogate biomarkers of seven plasma proteins, an estimate of smoking cigarette pack per year (these eight measures are components of GrimAge prediction), and an estimate of telomere length and predictions of blood cell counts. Table 1 provides a detailed list and description of DNAm-based measures that were used for statistical analysis in our work. Two subjects were filtered out in FM dataset as they had outlier values for DNAmAge estimate (values below Q1 – 1.5IQR or above Q3 + 1.5IQR, where Q1 and Q3 are first and third quartile, respectively, and IQR refers to interquartile range), reducing the total number of analyzed samples to 44 (24 cases and 20 controls). No outlier was found in the case of HPS and MOH/EM cohorts and all samples were retained.

**Table 1:** List of variables calculated by the new DNA methylation age calculator available online at https://dnamage.genetics.ucla.edu/.

**Variable name**	**Variable description**
DNAmAge	DNAm age estimate based on methylation of 353 CpG sites described by Horvath (44)
DNAmAgeHannum	DNAm age estimate based on methylation of 71 CpG sites described by Hannum et al. (45)
DNAmAgeSkinBloodClock	DNAm age estimate (based on methylation of 391 CpG sites) for human fibroblasts, keratinocytes, buccal cells, endothelial cells, lymphoblastoid cells, skin, blood, and saliva samples; developed by Horvath (44)
DNAmPhenoAge	DNAm-based estimate of phenotypic age (38)
DNAmGrimAge	DNA methylation age model build on eight DNAm based measures (DNAmADM, DNAmB2M, DNAmCystatinC, DNAmGDF15, DNAmLeptin, DNAmPACKYRS, DNAmPAI1, DNAmTIMP1), chronological age and sex (54)
DNAmTL	DNAm-based estimate of telomere length (55)
DNAmADM	DNAm-based prediction of plasma levels of adrenomedullin—a vasodilator peptide hormone (55)
DNAmB2M	DNAm-based prediction of plasma levels of beta-2 microglobulin—a component of major histocompatibility complex class 1 (MHC I) molecular (54)
DNAmCystatinC	DNAm-based prediction of plasma levels of cystatin C or (cystatin 3)—formerly called gamma trace, post-gamma-globulin, or neuroendocrine basic polypeptide (54)
DNAmGDF15	DNAm-based prediction of plasma levels of GDF-15—growth differentiation factor 15 (54)
DNAmLeptin	DNAm-based prediction of plasma levels of leptin—a hormone pre-dominantly present in adipose cells (54)
DNAmPAI1	DNAm-based prediction of plasma levels of plasminogen activator inhibitor antigen type 1 (PAI-1)—the major inhibitor of tissue-type plasminogen activator and unokinase plasminogen activator (54)
DNAmTIMP1	DNAm-based prediction of plasma levels of TIMP-1 or TIMP metallopeptidase inhibitor 1—a tissue inhibitor of metallo-proteinases (54)
DNAmPACKYRS	DNAm-based prediction of a number of pack of cigarettes during year (54)
CD8T	DNAm-based estimate of CD8 T cells, expressed as ordinal abundance measures (56)
CD4T	DNAm-based estimate of CD4 T cells, expressed as ordinal abundance measures (56)
CD8.naive	DNAm-based estimate of naive CD8 T cells, expressed as ordinal abundance measures (57, 58)
CD4.naive	DNAm-based estimate of naive CD4 T cells, expressed as ordinal abundance measures (57, 58)
CD8pCD28nCD45RAn	DNAm-based estimate of exhausted cytotoxic T defined as CD8+, CD28–, and CD45R– cells, expressed as ordinal abundance measures (57, 58)
NK	DNAm-based estimate of natural killer cells, expressed as ordinal abundance measures (56)
Bcell	DNAm-based estimate of B cells, expressed as ordinal abundance measures (56)
Mono	DNAm-based estimate of monocytes, expressed as ordinal abundance measures (56)
Gran	DNAm-based estimate of granulocytes, expressed as ordinal abundance measures (56)
PlasmaBlast	DNAm-based estimate of plasma blasts, expressed as ordinal abundance measures (57, 58)

### Statistical Analysis

Different methods of calculating biological age acceleration have been applied so far (59). Multiple linear regression (MLR) has been used to examine the influence of the disease status on DNAm age, correcting for chronological age and additional potential confounders. Alternatively, comparison of residuals of DNAm age regressed on chronological age (two-stage residual-outcome regression analysis, 2SR) has been largely used, although in genetic association studies, it has been shown that this method can lead to bias (60), and this could be true also in the case of epigenetics. To achieve consistent results, in this work, we have conducted parallel analyses and, for each of the epigenetic estimates listed in Table 1, we have compared the phenotypic groups using MLR or 2SR.

More specifically, in the first approach (MLR), the differences in each epigenetic variable among the phenotypic groups were examined building a linear regression model correcting for chronological age: *lm(Epigenetic_variable* ~ *Group* + *Age)*. For HPS twin cohort, the *lmer* function from the *lmerTest* R package was used to build a linear mixed model, including family as a random effect: *lmer(Epigenetic_variable* ~ *Group* + *Age* + *(1/Family))*.

In the second approach (2SR), each of the variables was adjusted for chronological age by building a linear regression model on the control group (healthy subjects in FM and MOH/EM cohorts, siblings with lower HPS in the HPS cohort)): *lm(Epigenetic_variable[control_group]* ~ *Age[control_group]*. This regression model was then applied to both cases and controls to predict the epigenetic variable under investigation and calculate the chronological age-corrected residuals. Finally, residuals were compared among the phenotypic groups using parametric Student's *t*-test, or paired Student's *t*-test in the case of HPS twin cohort.

Prior to hypothesis testing, the distribution of epigenetic variables was tested using the *ggqqplot* function in the *ggpubr* R package. According to visual inspection of the plots (data not shown), none of the variables violated the assumption of normality.

Power calculation for MLR and 2SR approaches was performed using the *pwr.t.test* function from the *pwr* R package (the *powerSim* function from *simr* R package was used for linear mixed models in the HPS cohort). As expected, given the small size of the cohorts, power tended to be low for most of the epigenetic variables; this was true especially for the 2SR approach, as previously reported (61).

Finally, we calculated the association between DNAm-based estimates and continuous clinical variables related to painful phenotypes, correcting for chronological age. In the HPS cohort, HPST values were considered and a linear mixed model was built, including family as a random effect: *lmer(Epigenetic_variable* ~ *HPST* + *Age* + *(1/Family))*. In the FM cohort, several clinical variables (duration of painful symptoms, MPQ, VAS, BPI FIQ, and PCS scores) were considered as follows: *lm(Epigenetic_variable* ~ *Clinical_variable* + *Age)*.

The results from all the analyses described above were corrected with Benjamini–Hochberg procedure for multiple tests: “locally”—within a single cohort and “globally”—within all the cohorts included in the study. The statistical significance level in all hypothesis tests was defined as α = 0.05.

All the analyses were conducted using R software (version 3.6.0 in Linux environment).

## Results

In our analysis, we considered three datasets of pain-related conditions: HPS, FM, and headache (MOH/EM). The characteristics of each dataset are summarized in Table S1. In each dataset, we analyzed a series of variables returned by the Horvath's epigenetic age calculator, including (1) different measures of epigenetic age (DNAmAge, DNAmAgeHannum, DNAmAgeSkinBloodClock, DNAmPhenoAge, GrimAge); (2) a DNAm-based estimate of telomere length (DNAmTL); (3) DNAm surrogates of components that contribute to GrimAge (abundance of adrenomedullin, DNAmADM; abundance of beta-2 microglobulin, DNAmB2M; abundance of cystatin C, DNAmCystatinC; abundance of growth differentiation factor 15, DNAmGDF15; abundance of leptin, DNAmLeptin; abundance of plasminogen activator inhibitor antigen type 1, DNAmPAI1; abundance of metallopeptidase inhibitor 1, DNAmTIMP1; predicted number of pack of cigarettes during year, DNAmPACKYRS); and (4) DNAm-based predictions of blood cell counts (CD8 T cells, CD8T; CD4 T cells, CD4T; naive CD8 T cells, CD8.naive; naive CD4 T cells, CD4.naive; joined estimation of CD8+, CD28–, and CD45RA– T cells, CD8pCD28nCD45RAn; natural killer cells, NK; B cells, Bcell; monocytes, Mono; granulocytes, Gran; plasma blasts, PlasmaBlast).

As described in the section Materials and Methods, we used two different approaches to compare the epigenetic variables listed above among the phenotypic groups within each dataset. In the first approach (MLR), we performed a MLR analysis correcting for chronological age. In the second approach (2SR), we compared the residuals of the epigenetic variable regressed on chronological age in control subjects within each dataset. Although the latter method has been largely used in the analysis of Horvath's clocks results, it has been associated to bias and loss of power in genetic association studies (60, 61). Accordingly, also in our datasets, the power was higher for the MLR approach compared with 2SR. For this reason, we provide the results of MLR in the main text and report those of the 2SR in Supplementary Materials.

### Heat Pain Sensitivity

**Figure 1:**
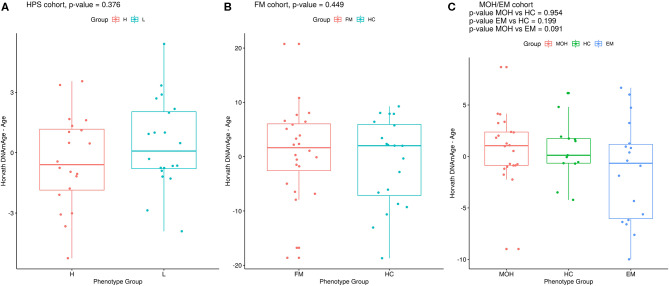
Epigenetic age difference (Horvath's DNAmAge – chronological age) adjusted for chronological age in the phenotypic groups in **(A)** HPS, **(B)** FM, and **(C)** MOH/EM cohorts. Reported *p-*values are from MLR analysis, as described in the Materials and Methods section.

Twenty monozygotic female twin pairs discordant for HPST were analyzed. The scatterplots of epigenetic estimates of age, DNAmGrimAge components, and blood cell counts against chronological age are reported respectively in Figures S1–S3. The results of the comparison between the twins with lower and higher HPST (using the MLR approach and including family as a random effect, see Materials and Methods) are reported in Table 2. No differences in epigenetic age acceleration were found between discordant twins (Figure 1A and Table 2). When considering nominal *p*-values, we found significant differences in estimates of CD8+ T blood cell counts (nominal *p* = 0.028; Table 2; Figure S3A): high pain sensitivity siblings showed decreased levels of CD8+ T cells compared with their co-twin. After correction for multiple tests, the difference in CD8+ T cells did not remain significant.

**Table 2:** Results of statistical hypothesis testing comparing discordant MZ twins with high and low heat pain sensitivity, using the MLR approach correcting for chronological age, and including family as a random effect.

**Epigenetic Variable**	**Coefficient**	***P*-value**	***P*-value LocAdjBH**	***P*-value GlobAdjBH**
DNAmAge	0.862	0.376	0.571	0.958
DNAmAgeHannum	1.779	0.163	0.449	0.942
DNAmAgeSkinBloodClock	0.789	0.381	0.571	0.958
DNAmPhenoAge	1.925	0.153	0.449	0.942
DNAmGrimAge	0.611	0.318	0.571	0.958
DNAmTL	−0.020	0.331	0.571	0.958
DNAmADM	4.194	0.168	0.449	0.942
DNAmB2M	14611.748	0.410	0.579	0.967
DNAmCystatinC	3930.380	0.377	0.571	0.958
DNAmGDF15	−43.823	0.150	0.449	0.942
DNAmLeptin	1124.843	0.313	0.571	0.958
DNAmPAI1	733.414	0.189	0.455	0.942
DNAmTIMP1	20.035	0.877	0.915	0.995
DNAmPACKYRS	0.710	0.613	0.736	0.995
CD8T	−0.022	**0.028**	0.449	0.942
CD4T	−0.002	0.902	0.915	0.995
CD8.naive	−0.612	0.915	0.915	0.995
CD4.naive	−29.158	0.072	0.449	0.942
CD8pCD28nCD45RAn	−0.592	0.451	0.601	0.967
NK	−0.006	0.597	0.736	0.995
Bcell	−0.009	0.118	0.449	0.942
Mono	0.002	0.703	0.803	0.995
Gran	0.033	0.098	0.449	0.942
PlasmaBlast	0.068	0.074	0.449	0.942

*The columns report the value of MLR coefficient (“Coefficient”), the corresponding nominal p-value (“P-value”), the p-value corrected with Benjamini–Hochberg procedure for multiple tests locally—within a single cohort (“P-value LocAdjBH”), and globally—within all the cohorts included in the study (“P-value GlobAdjBH”). Significant p-values are reported in bold*.

**Figure 2:**
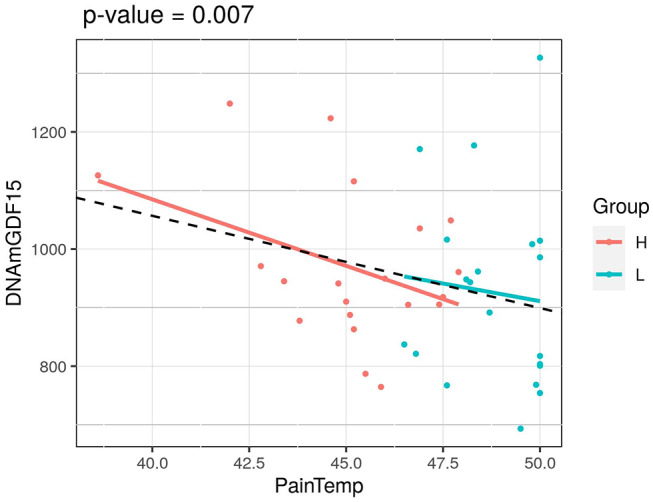
Association of HPST value and DNAmGDF15 in HPS cohort (L, twins with lower heat pain sensitivity; H, with higher heat pain sensitivity). The *p-*value of a mixed linear model correcting for age and using family as a random effect is reported.

We next considered the cohort as a whole, without dividing the twins according to HPST, and calculated the associations between HPST and epigenetic estimates using mixed model adjusted on age and including family as a random effect (Table 3). HPSTs were negatively associated with DNAmGDF15 (nominal *p* = 0.007; Table 3; Figure 2). After correction for multiple tests, this association was no longer significant.

**Table 3:** Results of association analysis between epigenetic measurements and HPST values in the HPS cohort, correcting for chronological age, and including family as a random effect.

**Epigenetic variable**	**Coefficient**	***P*-value**	***P*-value LocAdjBH**	***P*-value GlobAdjBH**
DNAmAge	0.038	0.872	0.947	0.995
DNAmAgeHannum	0.231	0.452	0.947	0.967
DNAmPhenoAge	0.135	0.677	0.947	0.995
DNAmAgeSkinBloodClock	0.093	0.671	0.947	0.995
DNAmGrimAge	0.143	0.332	0.947	0.958
DNAmADM	0.874	0.182	0.947	0.942
DNAmB2M	−882.527	0.831	0.947	0.995
DNAmCystatinC	452.914	0.648	0.947	0.995
DNAmGDF15	−16.463	**0.007**	0.159	0.757
DNAmLeptin	394.685	0.113	0.947	0.942
DNAmPAI1	190.268	0.126	0.947	0.942
DNAmTIMP1	−7.918	0.777	0.947	0.995
DNAmPACKYRS	0.243	0.483	0.947	0.991
DNAmTL	0.000	0.985	0.985	0.995
CD8T	−0.001	0.646	0.947	0.995
CD4T	0.001	0.807	0.947	0.995
CD8.naive	−0.163	0.908	0.947	0.995
CD4.naive	−4.764	0.246	0.947	0.958
CD8pCD28nCD45RAn	−0.153	0.417	0.947	0.967
NK	−0.001	0.750	0.947	0.995
Bcell	0.000	0.725	0.947	0.995
Mono	0.000	0.781	0.947	0.995
Gran	0.002	0.630	0.947	0.995
PlasmaBlast	0.007	0.389	0.947	0.958

*The columns report the value of regression coefficient (“Coefficient”), the corresponding nominal p-value (“P-value”), the p-value corrected with Benjamini–Hochberg procedure for multiple tests locally—within a single cohort (“P-value LocAdjBH”), and globally—within all the cohorts included in the study (“P-value GlobAdjBH”). Significant p-values are reported in bold*.

The subjects analyzed in the study by Cruz-Almeida et al. were older than 60 years. Thus, in order to make our results more comparable to those already published, we divided the HPS cohort in two subsets: twin pairs younger and older than 60 years old.

Twin pairs with age above 60 years old (12 couples) presented significant differences in DNAmAgeHannum age estimates (nominal *p* = 0.021; Table S2), and subjects with higher HPS were found to be epigenetically younger compared with their siblings. In the same subset, discordant twins differed in predicted CD8+ T and B cell counts (nominal *p* = 0.001 and 0.044, respectively; Table S2), with both estimates increased in more sensitive individuals. Only the difference in predicted CD8+ T cell counts was significant after correction for multiple tests (BH adjusted *p* = 0.033). No significant associations between epigenetic variables and HPST values were found in this subset (Table S3).

In the subset with subjects younger than 60 years old, DNAmGDF15 estimates were found to be significantly higher among siblings with lower HPST (nominal *p* = 0.026; Table S2). Association analysis confirmed negative relationship between HPST and DNAmGDF15 in this data subset (rnominal *p* = 0.002; Table S3). The latter association remained significant after multiple tests correction (BH adjusted *p* = 0.040).

The results obtained using 2SR approach were comparable to those presented above and are reported in Tables S4, S5.

The power analysis outcomes for MLR and 2SR approaches are reported in Tables S6, S7, respectively.

### Fibromyalgia

Twenty-four FM female cases and 20 sex- and age-matched controls that passed the quality control steps were analyzed. The scatterplots of epigenetic estimates of age, DNAmGrimAge components, and blood cell counts against chronological age are presented, respectively, in Figures S4–S6. In MLR analysis, we did not find differences in epigenetic age acceleration comparing FM patients and healthy subjects (Figure 1B and Table 4). The two phenotypic groups differed, however, in the ordinal abundance measure of naive CD4+ T cells adjusted by age, which was significantly lower, at the nominal level, in the affected individuals (nominal *p* = 0.025; Table 4; Figure S6D). The results obtained with 2SR approach were comparable to those of the MLR approach and are reported in Table S8.

**Table 4:** Results of statistical hypothesis testing comparing FM patients and healthy individuals (HC), using the MLR approach correcting for chronological age.

**Epigenetic variable**	**Coefficient**	***P*-value**	***P*-value LocAdjBH**	***P*-value GlobAdjBH**
DNAmAge	−2.168	0.449	0.963	0.967
DNAmAgeHannum	1.109	0.717	0.963	0.995
DNAmAgeSkinBloodClock	−0.662	0.814	0.963	0.995
DNAmPhenoAge	−0.971	0.736	0.963	0.995
DNAmGrimAge	−0.449	0.692	0.963	0.995
DNAmTL	0.030	0.676	0.963	0.995
DNAmADM	4.512	0.280	0.963	0.958
DNAmB2M	−4734.247	0.889	0.969	0.995
DNAmCystatinC	−10037.097	0.180	0.963	0.942
DNAmGDF15	−1.171	0.979	0.979	0.995
DNAmLeptin	−536.235	0.684	0.963	0.995
DNAmPAI1	−59.495	0.940	0.979	0.995
DNAmTIMP1	−47.894	0.843	0.963	0.995
DNAmPACKYRS	−1.560	0.649	0.963	0.995
CD8T	0.010	0.422	0.963	0.967
CD4T	0.009	0.610	0.963	0.995
CD8.naive	13.099	0.360	0.963	0.958
CD4.naive	67.771	**0.025**	0.599	0.942
CD8pCD28nCD45RAn	−0.330	0.753	0.963	0.995
NK	−0.017	0.171	0.963	0.942
Bcell	−0.006	0.415	0.963	0.967
Mono	0.002	0.750	0.963	0.995
Gran	−0.007	0.788	0.963	0.995
PlasmaBlast	0.047	0.361	0.963	0.958

*The columns report the value of MLR coefficient (“Coefficient”), the corresponding nominal p-value (“P-value”), the p-value corrected with Benjamini–Hochberg procedure for multiple tests locally—within a single cohort (“P-value LocAdjBH”), and globally—within all the cohorts included in the study (“P-value GlobAdjBH”). Significant p-values are reported in bold*.

**Figure 3:**
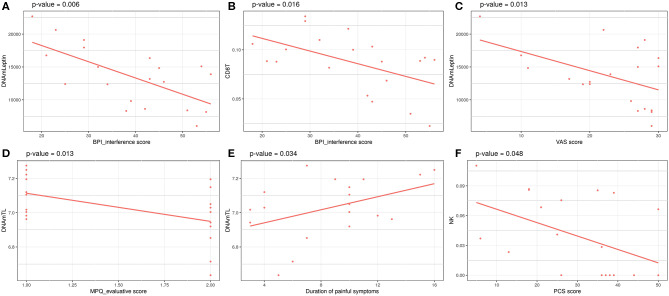
Significant associations between clinical data related to painful phenotype and epigenetic measurements in FM cohort: **(A)** BPI_interference score vs. DNAmLeptin estimates, **(B)** VAS score vs. DNAmLeptin estimates, **(C)** MPQ_evaluative score vs. DNAmTL, **(D)** BPI_interference score vs. CD8T estimates, **(E)** Duration of painful symptoms vs. DNAmTL estimates, **(F)** PCS score vs. NK cells estimates. *p-*values of a linear model correcting for age are reported.

Investigation of associations between a set of clinical data and the epigenetic estimates, correcting for chronological age, revealed significant negative association of BPI_interference with DNAmLeptin (nominal *p* = 0.006; Table 5; Figure 3A) and with predicted CD8+ T cell counts (nominal *p* = 0.016; Table 5; Figure 3B). The VAS score was also negatively associated with DNAmLeptin (nominal *p* = 0.013; Table 5; Figure 3C). MPQ_evaluative score was negatively associated with DNAmTL (nominal *p* = 0.013; Table 5; Figure 3D). Duration of painful symptoms expressed in years and DNAmTL were found to be positively associated (nominal *p* = 0.034; Table 5; Figure 3E). Finally, a negative association was found between PCS and abundance in NK cells (nominal *p* = 0.048; Table 5; Figure 3F). None of these associations remained significant after correction for multiple tests.

**Table 5:** Results of association analysis between epigenetic measurements and continuous clinical data related to phenotypes in FM cohort, correcting for chronological age.

**Clinical variable**	**Epigenetic variable**	**Coefficient**	***P*-value**	***P*-value LocAdjBH**	***P*-value GlobAdjBH**
BPI_interference	DNAmLeptin	−239.733	0.006	0.851	0.757
VAS	DNAmLeptin	−327.578	0.013	0.851	0.942
MPQ_evaluative	DNAmTL	−0.183	0.013	0.851	0.942
BPI_interference	CD8T	−0.002	0.016	0.851	0.942
Duration of painful symptoms	DNAmTL	0.022	0.034	0.992	0.942
PCS	NK	−0.001	0.048	0.992	0.942

*Only the associations with significant nominal p-values are reported. The columns report the value of the regression coefficient (“Coefficient”), the corresponding nominal p-value (“P-value”), the p-value corrected with Benjamini–Hochberg procedure for multiple tests locally—within a single cohort (“P-value LocAdjBH”), and globally—within all the cohorts included in the study (“P-value GlobAdjBH”)*.

The outcomes of power calculation for FM dataset are reported in Tables S9, S10.

### Headache

Twenty-two MOH patients, 18 EM cases, and 13 HC controls were analyzed. The scatterplots of epigenetic estimates of age, DNAmGrimAge components, and blood cell counts against chronological age are reported, respectively, in Figures S7–S9. MLR did not reveal any significant difference in epigenetic age acceleration, DNAm surrogates comprised in GrimAg, and estimates of telomere length and blood cell counts, between MOH and HC cases or between EM and HC cases (Figure 1C and Table 6). 2SR provided comparable results (Table S11).

**Table 6:** Results of statistical hypothesis testing comparing MOH patients, EM patients, and healthy individuals (HC), using the MLR approach correcting for chronological age.

	**MOH vs. HC**				**EM vs. HC**				**MOH vs. EM**			
**Epigenetic variable**	**Coefficient**	***P*-value**	***P*-value LocAdjBH**	***P*-value GlobAdjBH**	**Coefficient**	***P*-value**	***P*-value LocAdjBH**	***P*-value GlobAdjBH**	**Coefficient**	***P*-value**	***P*-value LocAdjBH**	***P*-value GlobAdjBH**
DNAmAge	0.066	0.954	0.968	0.995	2.157	0.199	0.606	0.942	2.354	0.091	0.721	0.942
DNAmAgeHannum	−1.708	0.378	0.824	0.958	1.975	0.313	0.751	0.958	0.701	0.712	0.912	0.995
DNAmAgeSkinBloodClock	0.539	0.610	0.915	0.995	0.023	0.986	0.986	0.995	1.009	0.331	0.721	0.958
DNAmPhenoAge	0.211	0.896	0.968	0.995	0.902	0.662	0.962	0.995	1.869	0.281	0.721	0.958
DNAmGrimAge	−0.785	0.583	0.915	0.995	1.598	0.175	0.606	0.942	0.855	0.493	0.845	0.994
DNAmTL	0.031	0.531	0.915	0.995	−0.007	0.903	0.985	0.995	0.011	0.798	0.912	0.995
DNAmADM	0.155	0.968	0.968	0.995	−1.918	0.678	0.962	0.995	−0.166	0.956	0.956	0.995
DNAmB2M	−35367.502	0.210	0.682	0.942	6664.817	0.832	0.962	0.995	−34406.624	0.195	0.721	0.942
DNAmCystatinC	−6991.375	0.227	0.682	0.945	353.046	0.953	0.986	0.995	−4276.995	0.349	0.721	0.958
DNAmGDF15	−8.906	0.860	0.968	0.995	10.909	0.842	0.962	0.995	−5.230	0.905	0.945	0.995
DNAmLeptin	−2140.873	0.297	0.771	0.958	2859.149	0.199	0.606	0.942	1191.950	0.361	0.721	0.958
DNAmPAI1	−1622.909	0.101	0.682	0.942	1178.540	0.389	0.847	0.958	−266.931	0.755	0.912	0.995
DNAmTIMP1	−79.089	0.542	0.915	0.995	−43.855	0.741	0.962	0.995	−57.791	0.672	0.912	0.995
DNAmPACKYRS	1.707	0.681	0.961	0.995	4.488	0.189	0.606	0.942	4.935	0.188	0.721	0.942
CD8T	−0.015	0.123	0.682	0.942	0.014	0.220	0.606	0.945	−0.002	0.845	0.922	0.995
CD4T	−0.003	0.792	0.968	0.995	0.023	0.206	0.606	0.942	0.014	0.337	0.721	0.958
CD8.naive	1.768	0.862	0.968	0.995	2.456	0.823	0.962	0.995	2.767	0.768	0.912	0.995
CD4.naive	43.989	0.146	0.682	0.942	13.125	0.680	0.962	0.995	45.008	0.105	0.721	0.942
CD8pCD28nCD45RAn	0.901	0.198	0.682	0.942	−1.173	0.117	0.606	0.942	−0.182	0.789	0.912	0.995
NK	−0.014	0.152	0.682	0.942	0.003	0.823	0.962	0.995	−0.009	0.309	0.721	0.958
Bcell	0.007	0.321	0.771	0.958	0.003	0.630	0.962	0.995	0.012	0.048	0.721	0.942
Mono	−0.004	0.427	0.853	0.967	−0.008	0.227	0.606	0.945	−0.008	0.120	0.721	0.942
Gran	0.030	0.106	0.682	0.942	−0.036	0.210	0.606	0.942	−0.008	0.723	0.912	0.995
PlasmaBlast	0.002	0.964	0.968	0.995	−0.039	0.424	0.847	0.967	−0.036	0.431	0.796	0.967

*The columns report the value of MLR coefficient (“Coefficient”), the corresponding nominal p-value (“P-value”), the p-value corrected with Benjamini–Hochberg procedure for multiple tests locally—within a single cohort (“P-value LocAdjBH”), and globally—within all the cohorts included in the study (“P-value GlobAdjBH”)*.

The outcomes of power calculation for MOH/EM dataset are reported in Tables S12, S13.

## Discussion

In this study, we analyzed methylation-based estimates of biological aging in three pain-related conditions, for which genome-wide DNAm data were available: HPS, FM, and medication overuse headache/episodic migraine (MOH/EM). In none of the three cohorts did we find evidences of epigenetic age acceleration associated to pain.

So far, only Cruz-Almeida et al. investigated the association between Horvath's epigenetic clock and chronic pain (46). The authors reported higher epigenetic age acceleration, expressed as difference between DNAmAge and chronological age, among 20 participants (age range: 60–83 years old) with persistent painful symptoms during the past 3 months compared with healthy age-matched controls. The study also showed significant negative partial correlations, accounting for age, sex, and race, between heat pain thresholds and epigenetic age. In a subsequent study, authors reported in the same cohort an association between brain age acceleration, predicted by structural neuroimaging, and chronic pain, but not with heat pain thresholds (62). It is worth to be noted that brain age acceleration was not observed in a similar group of chronic pain patients using any kind of pain remedies (63). This result suggests that the association between biomarkers of biological age and pain-related conditions is not obvious and that it can be modulated by several factors, including, for example, the use of medications (64). Thus, the differences between our results and those reported by Cruz-Almeida et al. (46) could be at least in part due to the different pain-related conditions evaluated. Furthermore, it should be noted that most of the subjects included in the FM and in the MOH/EM cohorts were younger than 60 years old, the lowest age in the cohort assessed by Cruz-Almeida et al. The HPS study included a larger number of subjects older than 60 years, but also when we considered this subset of twins, no age acceleration was observed in high pain sensitivity subjects.

It is worth to be noted that the HPS cohort does not involve a pathological phenotype, but is rather representative of differences naturally occurring within a population of individuals of different ages. Nevertheless, this cohort has been successfully used to identify epigenetic changes in the pain gene *TRPA1* [(47), p. 1], which have been confirmed in independent studies involving chronic pain patients (65). Several studies investigated if and how heat pain perception changes throughout the life (66–73), but they did not converge to consistent conclusions (67, 74, 75). In our analysis of HPS cohort, we observed a non-significant trend toward lower epigenetic ages in the high pain sensitivity twins. This trend was more marked after 60 years old, when age acceleration calculated by the DNAmAgeHannum predictor was significantly lower in the high pain sensitivity group. At the same time, when considering the cohort as a whole, we observed a non-significant trend toward an inverse association between epigenetic age acceleration (concordantly for all the five clocks) and HPST, similarly to what was observed by Cruz-Almeida et al. (62).

The second cohort that we considered includes female patients suffering from FM, one of the best-studied centralized pain conditions. As firstly proposed and summarized by Hassett et al. (76), FM patients show signs of premature aging, including a decrease in cognitive (77) and physical (78) condition, gray matter atrophy (79, 80) and a trend toward telomere shortening in leukocytes (81). In the latter study, subjects with higher pain intensities and more severe depression had shorter telomeres compared to milder phenotypes. In our cohort, on the contrary, no differences in DNAmTL were found between FM patients and healthy controls, and on the contrary, the duration of painful symptoms was positively associated with DNAmTL. One explanation for this observation is that patients experiencing painful symptoms for a longer time have also a longer history of medication use that can have attenuated age-associated telomere shortening, as previously suggested (82).

Finally, the third cohort that we considered in our study includes patients with MOH and EM. Also in this case, evidences in literature suggest the presence of age-related biological manifestations in the disease. Migraine patients tend to display thinner brain cortex compared with control subjects and this abnormal process seems to become more prominent with advanced chronological age (83, 84). Ren and colleagues reported significantly reduced telomere length among patients suffering from migraine compared with healthy controls (85), while a relationship between migraine and mitochondrial dysfunction has been largely described (86, 87).

Although our results do not provide evidence on acceleration of biological age expressed by epigenetic clocks, we identified a number of additional DNAm-based measures that are associated (mainly at the nominal level of significance) with pain-related phenotypes and that could reflect other alterations that are not captured by the clocks.

In the HPS dataset, we found higher age-adjusted estimates of blood CD8+ T cells counts in twins with high HPS compared with their siblings. This difference was more marked in the subgroup of subjects older than 60 years, where an increase in B cells was also observed. The reasons for this observation are unclear, but possibly related to a different inflammatory status of the co-twins. Changes in predicted blood cell counts were also found in the FM dataset, in which we observed a decrease in predicted CD4+ naive cells in patients and an inverse association between CD8+ T cells and NK cells and the severity of the disease symptoms, assessed as BPI_interference and PCS. Collectively, these results sustain the role of the immune system in pain-related conditions (88).

In the HPS cohort, HPST was negatively associated with DNAmGDF15 levels. Multiple reports showed that plasma levels of GDF15 increase with age (89, 90). Interestingly, GDF15 expression increased in dorsal spinal cord of rats with neuropathic pain (91) and higher serum levels of this protein were detected among myalgic encephalomyelitis/chronic fatigue syndrome patients when compared with healthy subjects (92). In the same study, GDF15 levels were shown to be positively associated with severity of disorder symptoms including fatigue and pain. Thus, our results support the hypothesis that increased levels of GDF15 could contribute to pain sensitivity.

Finally, increased DNAmLeptin levels were associated with less severe FM symptoms. Current data on leptin levels in pain-related conditions are controversial, possible due to high fluctuations in day-to-day leptin measurements (93). One study demonstrated that women with FM serum leptin levels are positively associated with the experience of pain (93). On the contrary, an independent study reported significantly reduced leptin levels in serum of Egyptian FM women compared with controls (94) and researches on animal models of nephropathies suggested that leptin may exert neuroprotective activity and bring pain relief (95–97).

In conclusion, in this paper, we investigated a set of DNAm estimates informative of biological age and of age-related parameters in different pain-related conditions. We did not find evidences of pain-related acceleration in epigenetic age, while we reported some changes in predicted blood cell counts and plasma protein levels. The main strength of our work is that it addresses a research question—the relationship between aging and chronic pain—which has been poorly investigated so far. We implemented a comprehensive approach to analyze age-related DNAm variables in various types of pain-related conditions. However, we are aware that our study has some limitations. The analyzed cohorts had small sample sizes and the statistical power tended to be low, possibly preventing to reach statistically significant results. Furthermore, the study missed replication datasets for each pain-related condition, on which the observed outcomes could be validated. Therefore, additional studies in independent cohorts are required to better characterize chronic pain conditions by epigenetic biomarkers of age.

## Data Availability Statement

The datasets analyzed for this study can be found in Gene Expression Omnibus (GEO) NCBI repository (http://www.ncbi.nlm.nih.gov/geo/) under accession numbers GSE53128 and GSE85506.

## Author Contributions

KK, PG, and CP contributed to the conception and design of the study. HK, DA, RT, GG, SC, GP, and PC organized the databases. KK, MB, and CS performed the statistical analysis. KK, MB, PG, and CP wrote the manuscript. All authors contributed to manuscript revision, read, and approved the submitted version.

## Conflict of Interest

The authors declare that the research was conducted in the absence of any commercial or financial relationships that could be construed as a potential conflict of interest.

## Supplementary Material

Figure S1 Associations between chronological age and DNAm-based biological age estimates in MZ twins discordant on heat pain sensitivity (L, twins with lower; H, with higher heat pain sensitivity): **(A)** DNAmAgeHorvath, **(B)** DNAmAgeHannum, **(C)** DNAmPhenoAge, **(D)** DNAmAgeSkinBloodClock, **(E)** DNAmGrimAge, **(F)** DNAmTL. *P*-values of linear regressions are reported for H and L twins separately.

Figure S2 Associations between chronological age and DNAm surrogates of components contributing to DNAmGrimAge in MZ twins discordant on heat pain sensitivity (L, twins with lower; H, with higher heat pain sensitivity): **(A)** DNAmADM, **(B)** DNAmB2M, **(C)** DNAmCystatinC, **(D)** DNAmGDF15, **(E)** DNAmLeptin, **(F)** DNAmPAI1, **(G)** DNAmTIMP1, **(H)** DNAmPACKYRS. *P*-values of linear regressions are reported for H and L twins separately.

Figure S3 Associations between chronological age and DNAm-based predictions of blood cell counts in MZ twins discordant on heat pain sensitivity (L, twins with lower; H with higher heat pain sensitivity): **(A)** CD8T, **(B)** CD4T, **(C)** CD8.naive, **(D)** CD4.naive, **(E)** CD8pCD28nCD45RAn, **(F)** NK, **(G)** Bcell, **(H)** Mono, **(I)** Gran, **(J)** PlasmaBlast. *P*-values of linear regressions are reported for H and L twins separately.

Figure S4 Associations between chronological age and DNAm-based biological age estimates in FM and HC samples: **(A)** DNAmAgeHorvath, **(B)** DNAmAgeHannum, **(C)** DNAmPhenoAge, **(D)** DNAmAgeSkinBloodClock, **(E)** DNAmGrimAge, **(F)** DNAmTL. *P*-values of linear regressions are reported for FM and HC samples.

Figure S5 Associations between chronological age and DNAm surrogates of components contributing to DNAmGrimAge in FM and HC samples: **(A)** DNAmADM, **(B)** DNAmB2M, **(C)** DNAmCystatinC, **(D)** DNAmGDF15, **(E)** DNAmLeptin, **(F)** DNAmPAI1, **(G)** DNAmTIMP1, **(H)** DNAmPACKYRS. *P*-values of linear regressions are reported for FM and HC samples.

Figure S6 Associations between chronological age and DNAm-based predictions of blood cell counts in FM and HC samples: **(A)** CD8T, **(B)** CD4T, **(C)** CD8.naive, **(D)** CD4.naive, **(E)** CD8pCD28nCD45RAn, **(F)** NK, **(G)** Bcell, **(H)** Mono, **(I)** Gran, **(J)** PlasmaBlast in FM cohort. *P*-values of linear regressions are reported for FM and HC samples.

Figure S7 Associations between chronological age and DNAm-based biological age estimates in MOH, EM and HC samples: **(A)** DNAmAgeHorvath, **(B)** DNAmAgeHannum, **(C)** DNAmPhenoAge, **(D)** DNAmAgeSkinBloodClock, **(E)** DNAmGrimAge, **(F)** DNAmTL. *P*-values of linear regressions are reported for MOH, EM and HC samples.

Figure S8 Associations between chronological age and DNAm surrogates of components contributing to DNAmGrimAge in MOH, EM and HC samples: **(A)** DNAmADM, **(B)** DNAmB2M, **(C)** DNAmCystatinC, **(D)** DNAmGDF15, **(E)** DNAmLeptin, **(F)** DNAmPAI1, **(G)** DNAmTIMP1, **(H)** DNAmPACKYRS in MOH/EM cohort. *P*-values of linear regressions are reported for MOH, EM and HC samples.

Figure S9 Associations between chronological age and DNAm-based predictions of blood cell counts in MOH, EM and HC samples: **(A)** CD8T, **(B)** CD4T, **(C)** CD8.naive, **(D)** CD4.naive, **(E)** CD8pCD28nCD45RAn, **(F)** NK, **(G)** Bcell, **(H)** Mono, **(I)** Gran, **(J)** PlasmaBlast in MOH/EM cohort. *P*-values of linear regressions are reported for MOH, EM and HC samples.
