# Supplementary figures and images for: Analysis of Epigenetic Age Predictors in Pain-Related Conditions
Source: Front Public Health. 2020 Jun 9;8:172. doi: 10.3389/fpubh.2020.00172 (PMC7296181; doi:10.3389/fpubh.2020.00172)

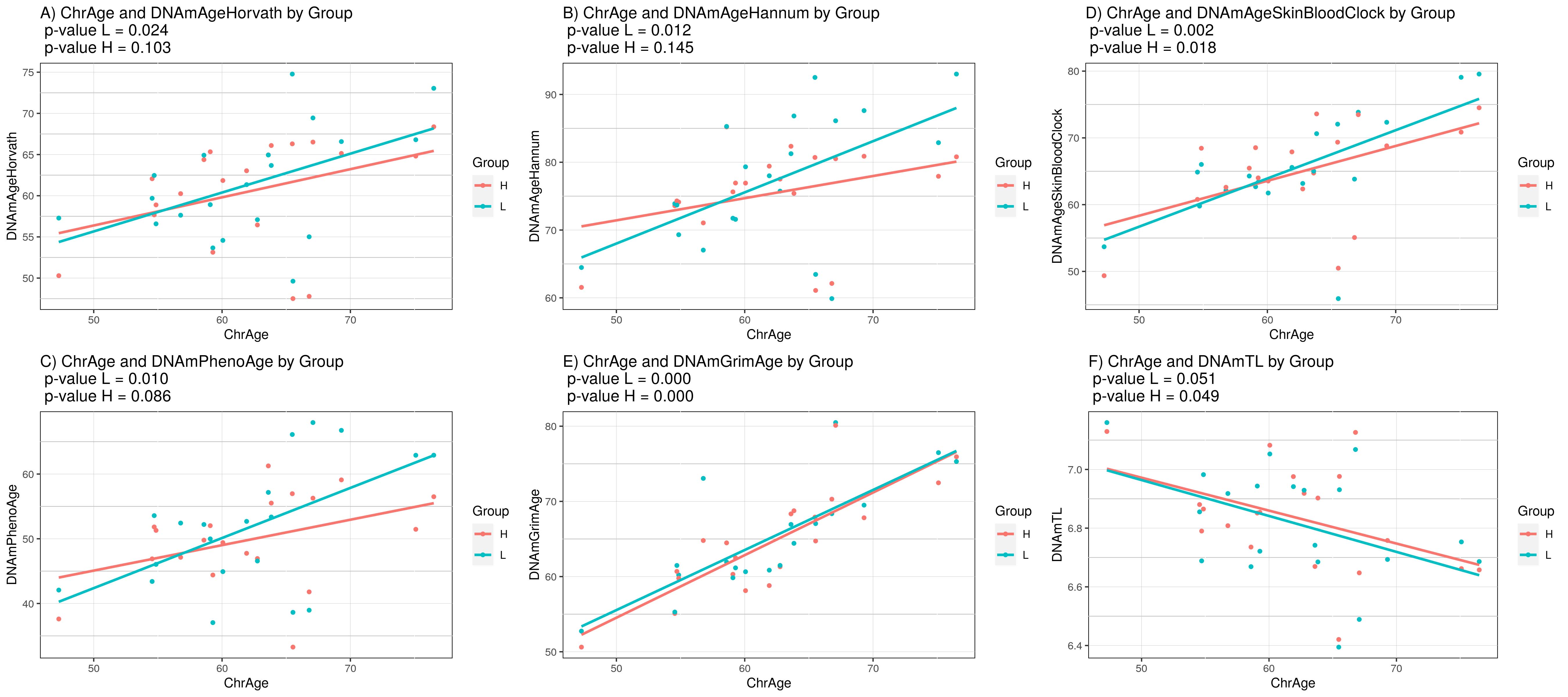

Supplement: Figure S1 — Associations between chronological age and DNAm-based biological age estimates in MZ twins discordant on heat pain sensitivity (L, twins with lower; H, with higher heat pain sensitivity): (A) DNAmAgeHorvath, (B) DNAmAgeHannum, (C) DNAmPhenoAge, (D) DNAmAgeSkinBloodClock, (E) DNAmGrimAge, (F) DNAmTL. P-values of linear regressions are reported for H and L twins separately. [file Image_1.JPEG]

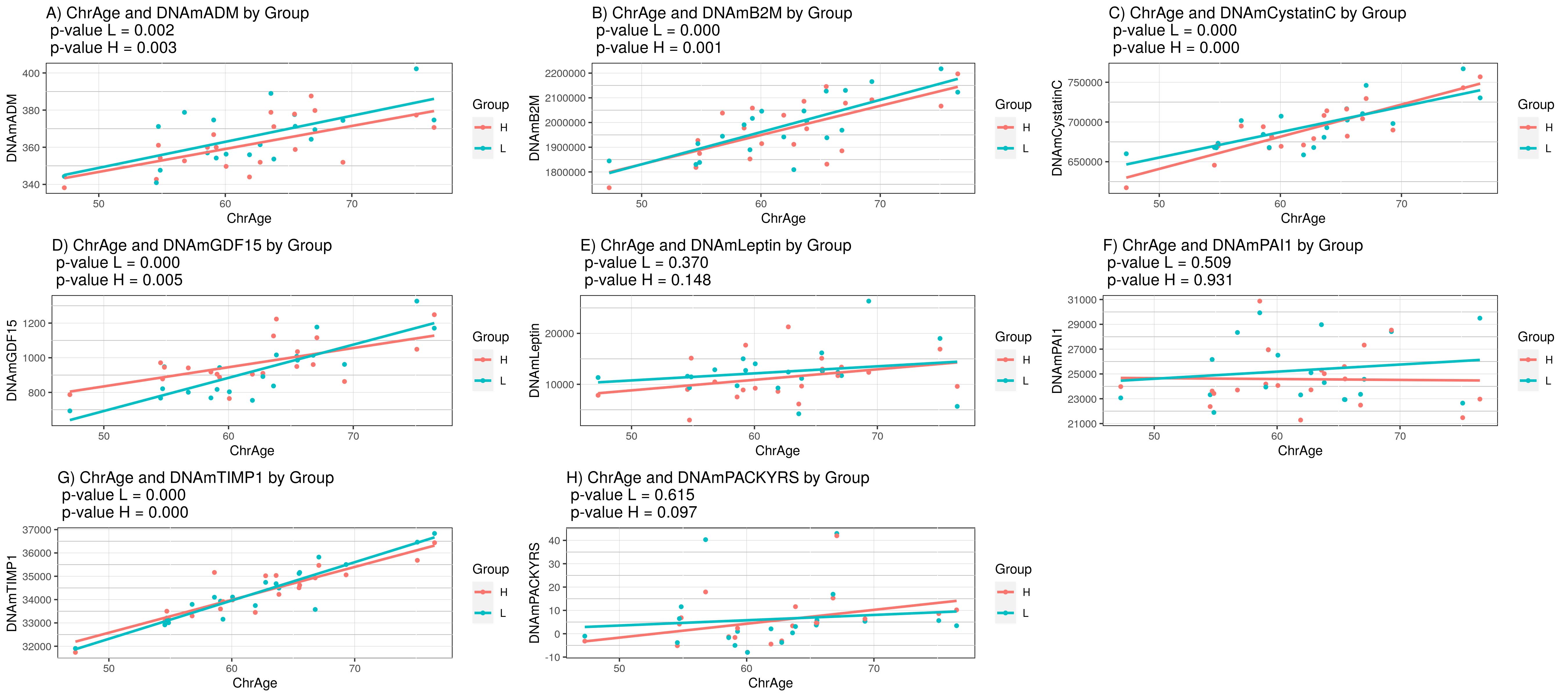

Supplement: Figure S2 — Associations between chronological age and DNAm surrogates of components contributing to DNAmGrimAge in MZ twins discordant on heat pain sensitivity (L, twins with lower; H, with higher heat pain sensitivity): (A) DNAmADM, (B) DNAmB2M, (C) DNAmCystatinC, (D) DNAmGDF15, (E) DNAmLeptin, (F) DNAmPAI1, (G) DNAmTIMP1, (H) DNAmPACKYRS. P-values of linear regressions are reported for H and L twins separately. [file Image_2.JPEG]

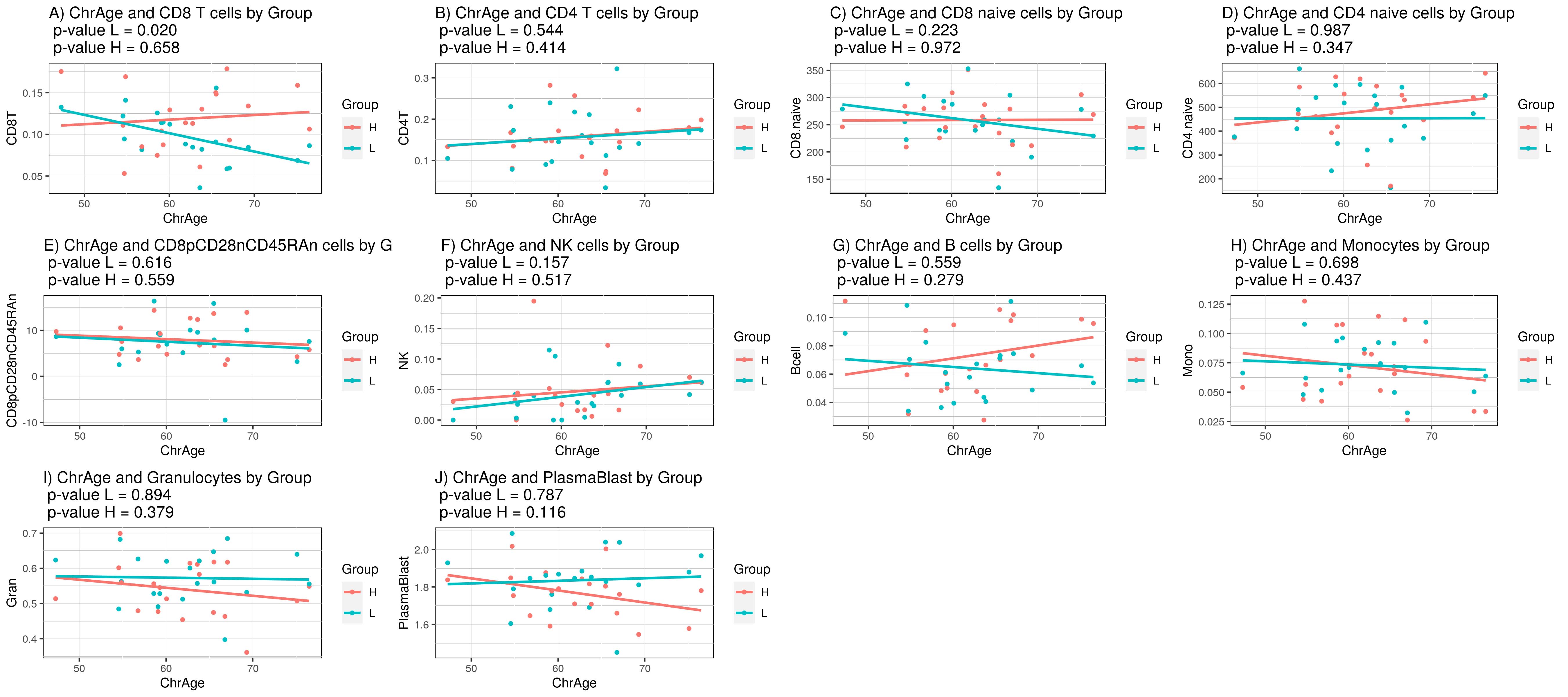

Supplement: Figure S3 — Associations between chronological age and DNAm-based predictions of blood cell counts in MZ twins discordant on heat pain sensitivity (L, twins with lower; H with higher heat pain sensitivity): (A) CD8T, (B) CD4T, (C) CD8.naive, (D) CD4.naive, (E) CD8pCD28nCD45RAn, (F) NK, (G) Bcell, (H) Mono, (I) Gran, (J) PlasmaBlast. P-values of linear regressions are reported for H and L twins separately. [file Image_3.JPEG]

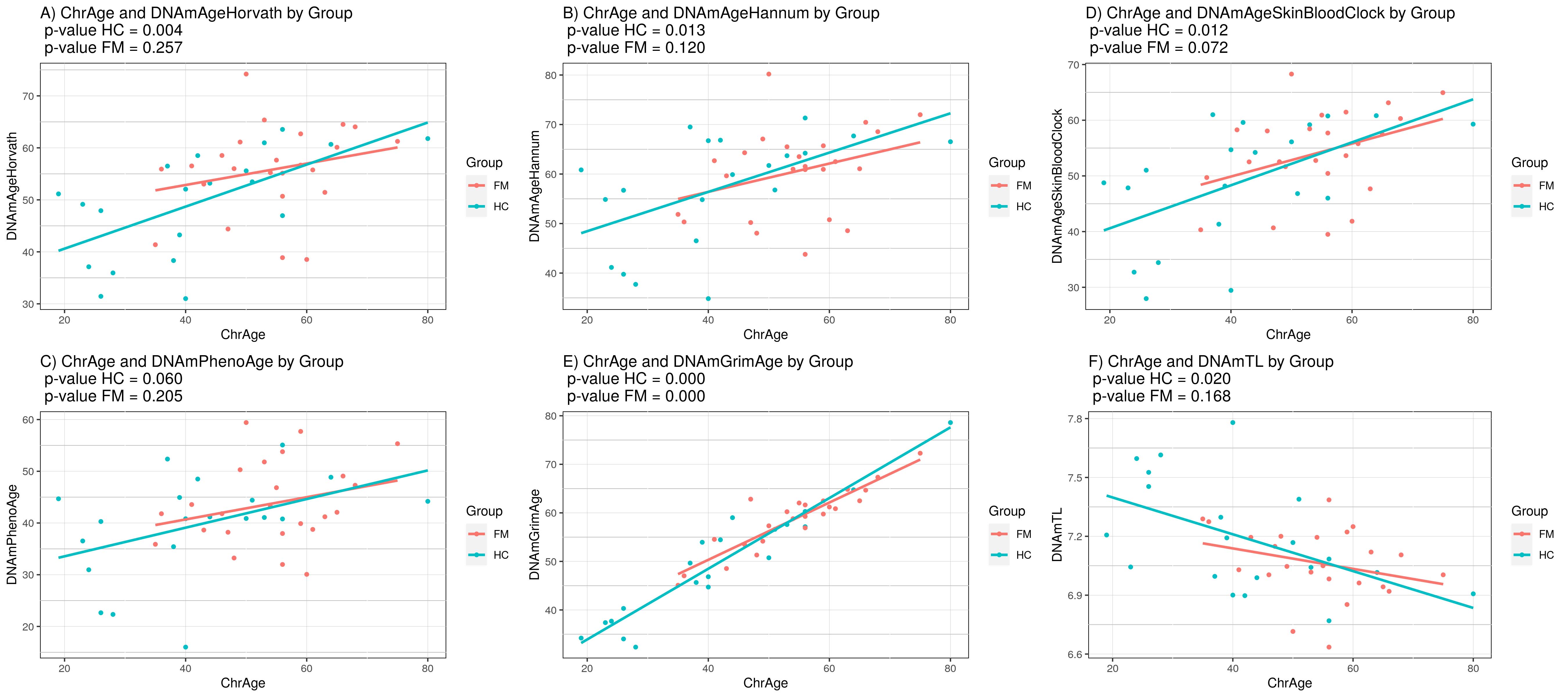

Supplement: Figure S4 — Associations between chronological age and DNAm-based biological age estimates in FM and HC samples: (A) DNAmAgeHorvath, (B) DNAmAgeHannum, (C) DNAmPhenoAge, (D) DNAmAgeSkinBloodClock, (E) DNAmGrimAge, (F) DNAmTL. P-values of linear regressions are reported for FM and HC samples. [file Image_4.JPEG]

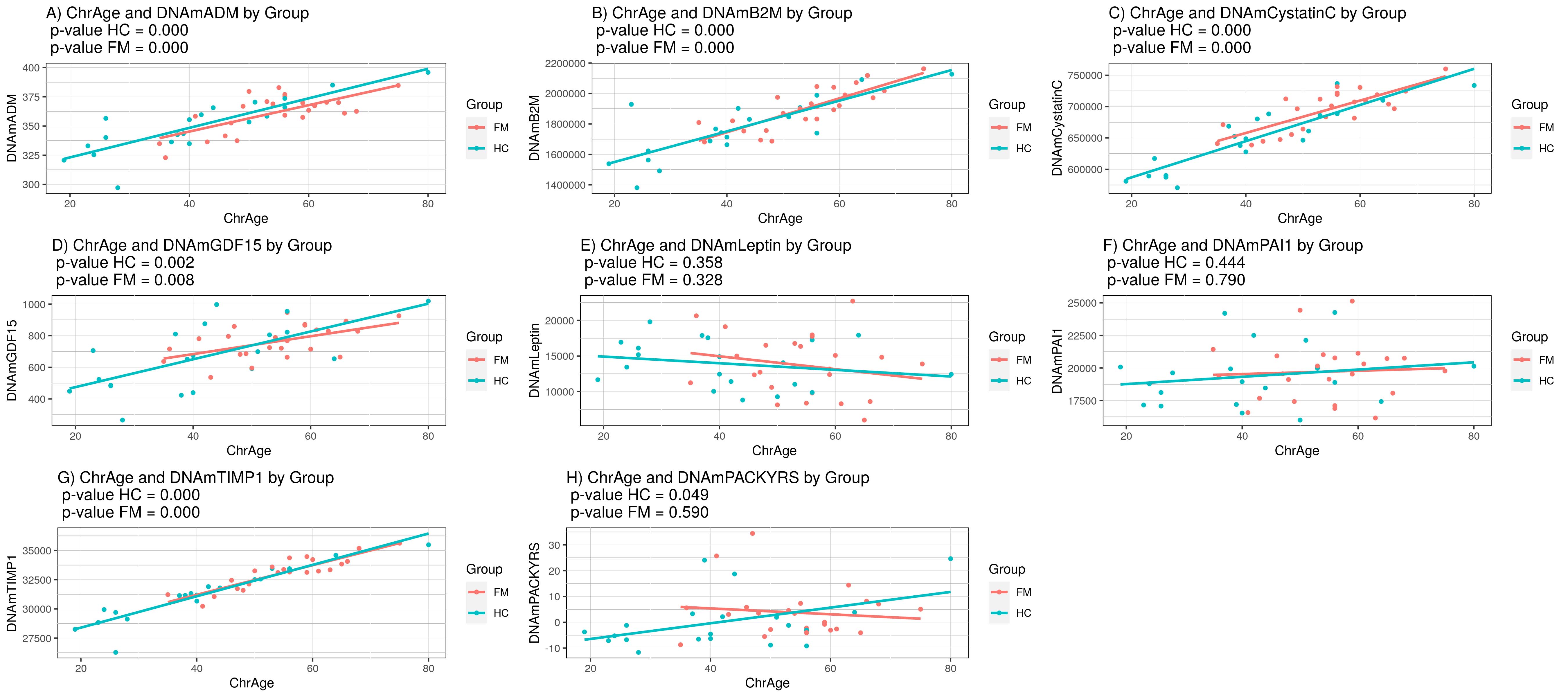

Supplement: Figure S5 — Associations between chronological age and DNAm surrogates of components contributing to DNAmGrimAge in FM and HC samples: (A) DNAmADM, (B) DNAmB2M, (C) DNAmCystatinC, (D) DNAmGDF15, (E) DNAmLeptin, (F) DNAmPAI1, (G) DNAmTIMP1, (H) DNAmPACKYRS. P-values of linear regressions are reported for FM and HC samples. [file Image_5.JPEG]

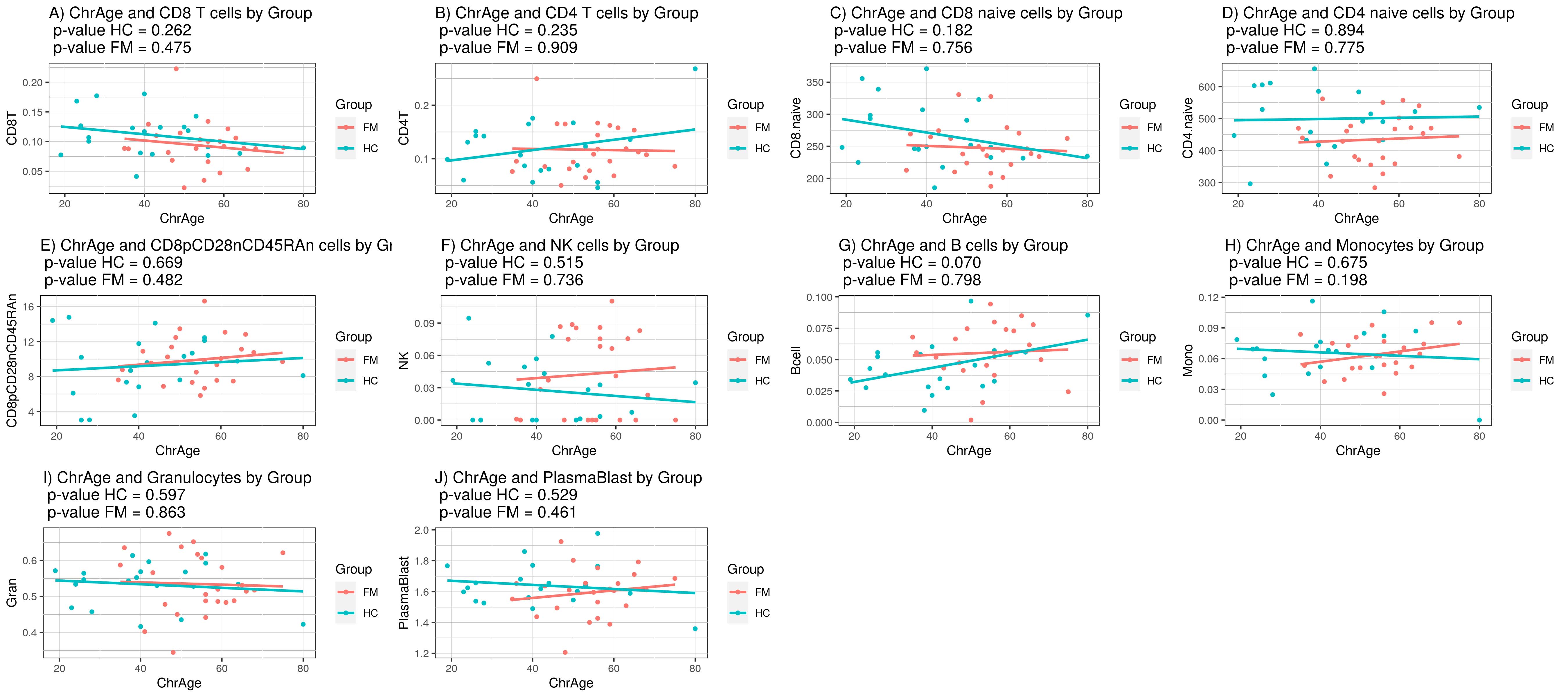

Supplement: Figure S6 — Associations between chronological age and DNAm-based predictions of blood cell counts in FM and HC samples: (A) CD8T, (B) CD4T, (C) CD8.naive, (D) CD4.naive, (E) CD8pCD28nCD45RAn, (F) NK, (G) Bcell, (H) Mono, (I) Gran, (J) PlasmaBlast in FM cohort. P-values of linear regressions are reported for FM and HC samples. [file Image_6.JPEG]

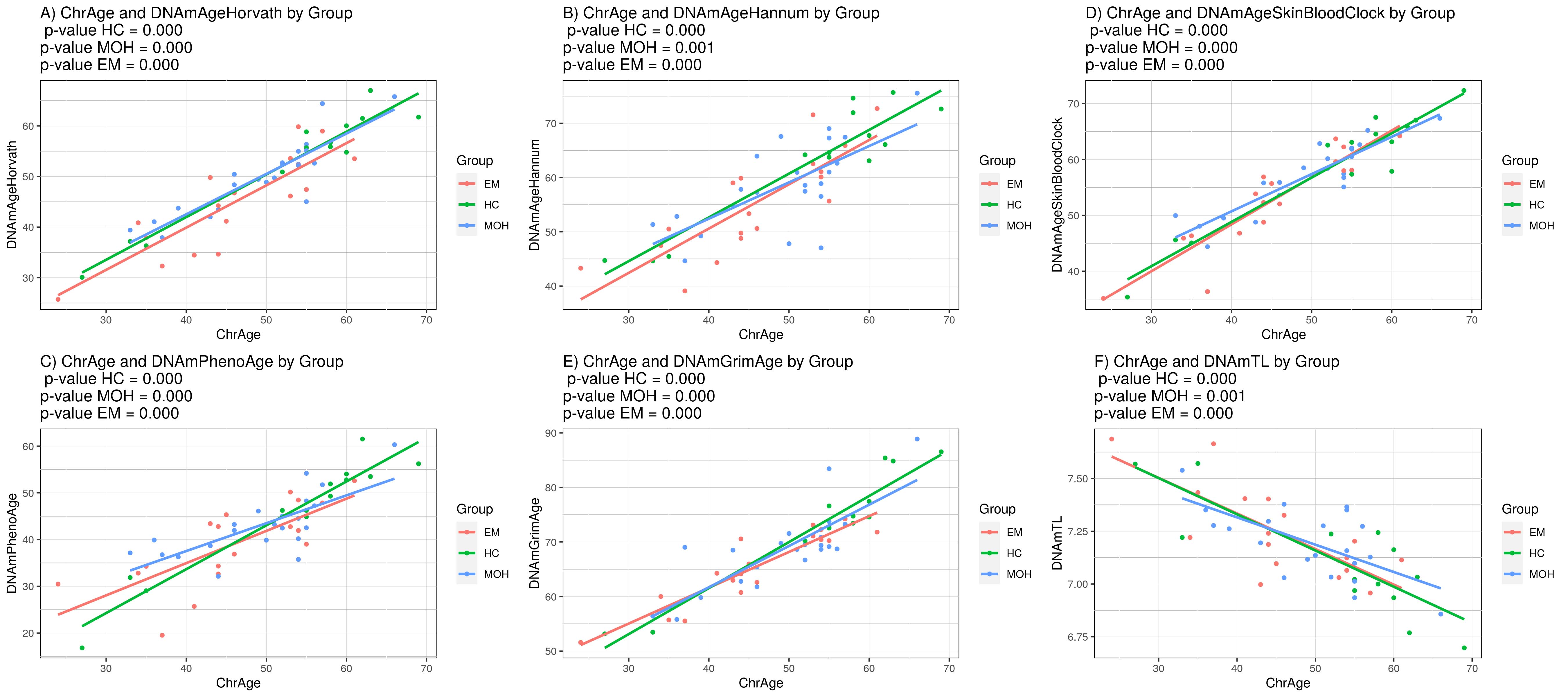

Supplement: Figure S7 — Associations between chronological age and DNAm-based biological age estimates in MOH, EM and HC samples: (A) DNAmAgeHorvath, (B) DNAmAgeHannum, (C) DNAmPhenoAge, (D) DNAmAgeSkinBloodClock, (E) DNAmGrimAge, (F) DNAmTL. P-values of linear regressions are reported for MOH, EM and HC samples. [file Image_7.JPEG]

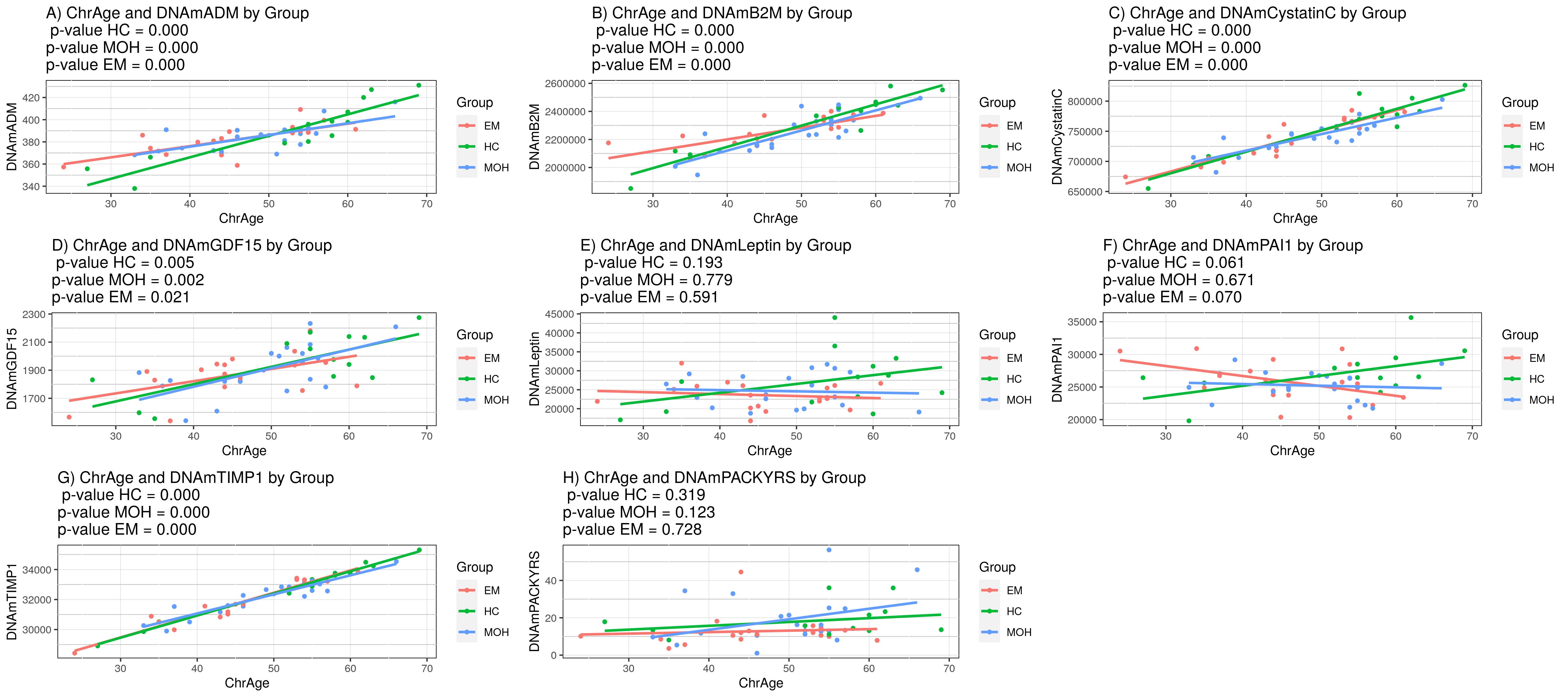

Supplement: Figure S8 — Associations between chronological age and DNAm surrogates of components contributing to DNAmGrimAge in MOH, EM and HC samples: (A) DNAmADM, (B) DNAmB2M, (C) DNAmCystatinC, (D) DNAmGDF15, (E) DNAmLeptin, (F) DNAmPAI1, (G) DNAmTIMP1, (H) DNAmPACKYRS in MOH/EM cohort. P-values of linear regressions are reported for MOH, EM and HC samples. [file Image_8.JPEG]

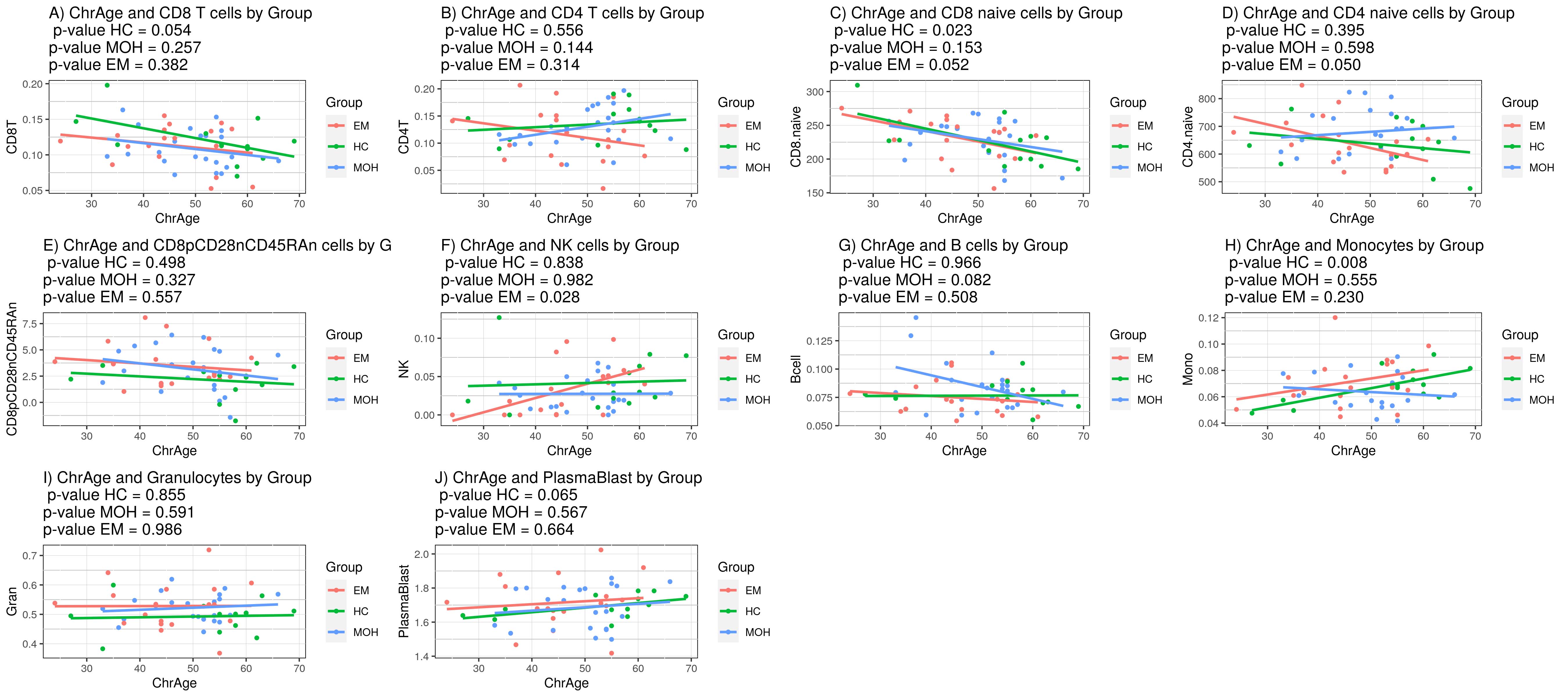

Supplement: Figure S9 — Associations between chronological age and DNAm-based predictions of blood cell counts in MOH, EM and HC samples: (A) CD8T, (B) CD4T, (C) CD8.naive, (D) CD4.naive, (E) CD8pCD28nCD45RAn, (F) NK, (G) Bcell, (H) Mono, (I) Gran, (J) PlasmaBlast in MOH/EM cohort. P-values of linear regressions are reported for MOH, EM and HC samples. [file Image_9.JPEG]
