# Supplementary material for: Analysis of Epigenetic Age Predictors in Pain-Related Conditions
Source: Front Public Health. 2020 Jun 9;8:172. doi: 10.3389/fpubh.2020.00172 (PMC7296181; doi:10.3389/fpubh.2020.00172)
Supplement: Supplementary file 10 [file Data_Sheet_1.PDF]

Supplementary Tables

Table S1. DNA methylation studies used for the analysis of epigenetic age estimates in pain-related conditions.

Cohort	Pain-related condition	Size	Phenotypes		Age ± sd	Age range	Country	Technology	Reference
HPS	Heat Pain Sensitivity (HPS)	40	20 monozygotic twin pairs discordant for heat pain temperature	40 females	62.15 ± 6.98	47 -76	United Kingdom	450k	(Bell et al. 2014)
FM	Fibromyalgia (FM)	44	24 FM	24 females	54.21 ± 9.99	35 - 75	Brazil	450k	(Ciampi de Andrade et al. 2017)
			20 HC	20 females	41.80 ± 15.51	19 - 80			
MOH/EM	Medication-Overuse Headache (MOH) and Episodic Migraine (EM)	53	22 MOH	20 females<br>2 males	49.27 ± 8.03	33 - 66	Italy	EPIC	(Terlizzi et al. 2018)
			18 EM	17 females<br>1 male	52.85 ± 12.89	27 - 69			
			13 HC	8 females<br>5 males	45.78 ± 9.48	24 - 61			

References

Bell, J. T., A. K. Loomis, L. M. Butcher, F. Gao, B. Zhang, C. L. Hyde, J. Sun, et al. 2014. “Differential Methylation of the TRPA1 Promoter in Pain Sensitivity.” *Nature Communications* 5: 2978. https://doi.org/10.1038/ncomms3978.

Ciampi de Andrade, Daniel, Mariana Maschietto, Ricardo Galhardoni, Gisele Gouveia, Thais Chile, Ana C. Victorino Krepischi, Camila S. Dale, et al. 2017. “Epigenetics Insights into Chronic Pain: DNA Hypomethylation in Fibromyalgia—a Controlled Pilot-Study.” *PAIN* 158 (8): 1473. https://doi.org/10.1097/j.pain.0000000000000932.

Terlizzi, Rossana, Maria Giulia Bacalini, Chiara Pirazzini, Giulia Giannini, Giulia Pierangeli, Paolo Garagnani, Claudio Franceschi, Sabina Cevoli, and Pietro Cortelli. 2018. “Epigenetic DNA Methylation Changes in Episodic and Chronic Migraine.” *Neurological Sciences: Official Journal of the Italian Neurological Society and of the Italian Society of Clinical Neurophysiology* 39 (Suppl 1): 67–68. https://doi.org/10.1007/s10072-018-3348-8.

Table S2. Results of statistical hypothesis testing comparing discordant MZ twins with high and low heat pain sensitivity, analyzing separately individuals with age below and above 60 years. In both the subsets, we used the MLR approach correcting for chronological age and including family as a random effect. The columns report respectively: the value of MLR coefficient ("Coefficient"), the corresponding nominal p-value ("P-value"), the p-value corrected with Benjamini-Hochberg procedure for multiple tests locally - within a single cohort ("P-value LocAdjBH"), and globally - within all the cohorts included in the study ("P-value GlobAdjBH"). Significant p-values are reported in bold.

	ABOVE 60 YEARS				BELOW 60 YEARS			
**Epigenetic Variable**	**Coefficient**	**P-value**	**P-value LocAdjBH**	**P-value GlobAdjBH**	**Coefficient**	**P-value**	**P-value LocAdjBH**	**P-value GlobAdjBH**
DNAmAge	1.508	0.246	0.541	0.958	-0.107	0.946	0.947	0.995
DNAmAgeHannum	4.247	**0.021**	0.247	0.942	-1.923	0.112	0.947	0.942
DNAmAgeSkinBloodClock	1.505	0.248	0.541	0.958	-0.286	0.814	0.947	0.995
DNAmPhenoAge	3.559	0.066	0.308	0.942	-0.526	0.759	0.947	0.995
DNAmGrimAge	0.398	0.560	0.747	0.995	0.930	0.449	0.947	0.967
DNAmTL	-0.036	0.181	0.482	0.942	0.003	0.936	0.947	0.995
DNAmADM	4.238	0.344	0.550	0.958	4.129	0.321	0.947	0.958
DNAmB2M	25476.165	0.338	0.550	0.958	-1684.878	0.939	0.947	0.995
DNAmCystatinC	1215.900	0.847	0.847	0.995	8002.098	0.219	0.947	0.945
DNAmGDF15	-16.929	0.706	0.771	0.995	-84.164	**0.026**	0.617	0.942
DNAmLeptin	668.080	0.677	0.771	0.995	1809.987	0.255	0.947	0.958
DNAmPAI1	847.306	0.298	0.550	0.958	562.576	0.471	0.947	0.980
DNAmTIMP1	152.907	0.380	0.570	0.958	-179.272	0.371	0.947	0.958
DNAmPACKYRS	-1.135	0.340	0.550	0.958	3.478	0.261	0.947	0.958
CD8T	-0.042	**0.001**	**0.033**	0.377	0.008	0.498	0.947	0.995
CD4T	0.004	0.828	0.847	0.995	-0.010	0.549	0.947	0.995
CD8.naive	-10.571	0.109	0.328	0.942	14.327	0.146	0.947	0.942
CD4.naive	-38.308	0.058	0.308	0.942	-15.434	0.597	0.947	0.995
CD8pCD28nCD45RAn	-0.892	0.476	0.672	0.983	-0.140	0.850	0.947	0.995
NK	-0.005	0.658	0.771	0.995	-0.008	0.750	0.947	0.995
Bcell	-0.016	**0.044**	0.308	0.942	0.002	0.799	0.947	0.995
Mono	0.003	0.657	0.771	0.995	0.000	0.947	0.947	0.995
Gran	0.047	0.086	0.308	0.942	0.012	0.696	0.947	0.995
PlasmaBlast	0.096	0.090	0.308	0.942	0.026	0.589	0.947	0.995

Table S3. Results of association analysis between epigenetic measurements and HPST values in HPS cohort, analyzing separately individuals with age below and above 60 years, correcting for chronological age and including family as a random effect.

	ABOVE 60 YEARS				BELOW 60 YEARS			
**Epigenetic Variable**	**Coefficient**	**P-value**	**P-value LocAdjBH**	**P-value GlobAdjBH**	**Coefficient**	**P-value**	**P-value LocAdjBH**	**P-value GlobAdjBH**
DNAmAge	0.143	0.650	0.993	0.995	0.003	0.994	0.994	0.995
DNAmAgeHannum	0.723	0.101	0.989	0.942	-0.466	0.123	0.593	0.942
DNAmAgeSkinBloodClock	0.358	0.447	0.993	0.967	-0.105	0.802	0.994	0.995
DNAmPhenoAge	0.174	0.582	0.993	0.995	-0.075	0.797	0.994	0.995
DNAmGrimAge	0.021	0.897	0.993	0.995	0.365	0.213	0.653	0.942
DNAmTL	-0.003	0.606	0.993	0.995	0.007	0.440	0.994	0.967
DNAmADM	0.611	0.507	0.993	0.995	1.277	0.186	0.653	0.942
DNAmB2M	-820.552	0.892	0.993	0.995	-1452.762	0.788	0.994	0.995
DNAmCystatinC	-474.249	0.724	0.993	0.995	2266.437	0.121	0.593	0.942
DNAmGDF15	-12.447	0.151	0.989	0.942	-22.825	**0.002**	**0.040**	0.377
DNAmLeptin	417.894	0.237	0.989	0.958	310.221	0.395	0.994	0.961
DNAmPAI1	70.685	0.681	0.993	0.995	237.034	0.218	0.653	0.945
DNAmTIMP1	-11.500	0.757	0.993	0.995	-5.630	0.905	0.994	0.995
DNAmPACKYRS	-0.337	0.247	0.989	0.958	1.156	0.116	0.593	0.942
CD8T	0.000	0.972	0.993	0.995	0.001	0.720	0.994	0.995
CD4T	0.001	0.882	0.993	0.995	0.000	0.948	0.994	0.995
CD8.naive	-2.844	0.071	0.989	0.942	3.735	0.124	0.593	0.942
CD4.naive	-7.097	0.166	0.989	0.942	-1.007	0.892	0.994	0.995
CD8pCD28nCD45RAn	-0.281	0.340	0.993	0.958	-0.044	0.813	0.994	0.995
NK	0.001	0.741	0.993	0.995	-0.003	0.544	0.994	0.995
Bcell	0.000	0.971	0.993	0.995	0.001	0.541	0.994	0.995
Mono	0.000	0.993	0.993	0.995	0.000	0.784	0.994	0.995

Table S4. Results of statistical hypothesis testing comparing discordant MZ twins with high and low heat pain sensitivity, using the 2SR approach. For each variable, mean and median values of residuals in the two groups are reported. Significant p-values are reported in bold.

	AVERAGE(Age-adj)			MEDIAN(Age-adj)	
**Variable Name**	**High Sensitivity**	**Low Sensitivity**	**P-value**	**High Sensitivity**	**Low Sensitivity**
DNAmAge	-0.862	0.000	0.376	1.160	0.765
DNAmAgeHannum	-1.779	0.000	0.163	0.324	1.558
DNAmAgeSkinBloodClock	-0.789	0.000	0.381	0.205	0.403
DNAmPhenoAge	-1.925	0.000	0.153	0.095	0.833
DNAmGrimAge	-0.611	0.000	0.318	-0.627	-0.763
DNAmTL	0.020	0.000	0.331	0.005	0.042
DNAmADM	-4.194	0.000	0.168	-3.828	-3.690
DNAmB2M	-14612.000	0.000	0.410	-5028.000	23743.000
DNAmCystatinC	-3930.000	0.000	0.377	-2175.000	-2168.000
DNAmGDF15	43.820	0.000	0.150	46.850	-8.287
DNAmLeptin	-1124.800	0.000	0.313	-1894.500	-49.850
DNAmPAI1	-733.410	0.000	0.190	-1032.200	-1061.000
DNAmTIMP1	-20.040	0.000	0.877	-132.010	120.470
DNAmPACKYRS	-0.710	0.000	0.613	-2.428	-3.753
CD8T	0.022	0.000	**0.028**	0.023	0.002
CD4T	0.001	0.000	0.902	-0.001	-0.007
CD8.naive	0.612	0.000	0.915	4.202	-2.844
CD4.naive	29.160	0.000	0.072	32.930	28.040
CD8pCD28nCD45RAn	0.592	0.000	0.451	-0.412	0.295
NK	0.006	0.000	0.597	-0.001	-0.007
Bcell	0.009	0.000	0.119	0.005	0.000
Mono	-0.002	0.000	0.703	-0.008	-0.002
Gran	-0.033	0.000	0.098	-0.024	-0.011
PlasmaBlast	-0.068	0.000	0.074	-0.062	0.016

Table S5. Results of statistical hypothesis testing comparing discordant MZ twins with high and low heat pain sensitivity, analyzing separately individuals with age above and below 60 years, and using the 2SR approach. For each variable and for each subset, mean values of residuals in the two groups are reported. Significant p-values are reported in bold.

	ABOVE 60 YEARS			BELOW 60 YEARS		
	**AVERAGE(Age-adj)**			**AVERAGE(Age-adj)**		
**Variable Name**	**High Sensitivity**	**Low Sensitivity**	**P-value**	**High Sensitivity**	**Low Sensitivity**	**P-value**
DNAmAge	-1.508	0.000	0.246	0.107	0.000	0.946
DNAmAgeHannum	-4.247	0.000	**0.021**	1.923	0.000	0.112
DNAmAgeSkinBloodClock	-1.506	0.000	0.248	0.286	0.000	0.814
DNAmPhenoAge	-3.559	0.000	0.066	0.526	0.000	0.759
DNAmGrimAge	-0.398	0.000	0.560	-0.930	0.000	0.449
DNAmTL	0.036	0.000	0.181	-0.003	0.000	0.936
DNAmADM	-4.238	0.000	0.344	-4.129	0.000	0.321
DNAmB2M	-25476.000	0.000	0.338	1685.000	0.000	0.939
DNAmCystatinC	-1216.000	0.000	0.847	-8002.000	0.000	0.219
DNAmGDF15	16.929	0.000	0.706	84.160	0.000	**0.026**
DNAmLeptin	-668.100	0.000	0.677	-1810.000	0.000	0.255
DNAmPAI1	-847.300	0.000	0.298	-562.600	0.000	0.471
DNAmTIMP1	-152.900	0.000	0.380	179.270	0.000	0.371
DNAmPACKYRS	1.135	0.000	0.341	-3.478	0.000	0.261
CD8T	0.042	0.000	**0.001**	-0.008	0.000	0.498
CD4T	-0.004	0.000	0.828	0.010	0.000	0.549
CD8.naive	10.570	0.000	0.109	-14.327	0.000	0.146
CD4.naive	38.310	0.000	0.058	15.434	0.000	0.597
CD8pCD28nCD45RAn	0.892	0.000	0.476	0.140	0.000	0.850
NK	0.005	0.000	0.658	0.008	0.000	0.750
Bcell	0.016	0.000	**0.044**	-0.002	0.000	0.799
Mono	-0.003	0.000	0.657	0.000	0.000	0.947
Gran	-0.047	0.000	0.086	-0.012	0.000	0.696
PlasmaBlast	-0.096	0.000	0.090	-0.026	0.000	0.589

Table S6. Results of power calculation for MLR in HPS cohort (number of simulations = 1000).

	**Power of the study with default effect size**		
**Epigenetic Variable**	ALL HPS SAMPLES	ABOVE 60 YEARS	BELOW 60 YEARS
DNAmAge	0.150	0.240	0.070
DNAmAgeHannum	0.300	0.750	0.440
DNAmAgeSkinBloodClock	0.160	0.230	0.100
DNAmPhenoAge	0.350	0.520	0.100
DNAmGrimAge	0.190	0.140	0.150
DNAmTL	0.190	0.290	0.080
DNAmADM	0.310	0.200	0.250
DNAmB2M	0.140	0.180	0.080
DNAmCystatinC	0.150	0.070	0.300
DNAmGDF15	0.320	0.090	0.830
DNAmLeptin	0.160	0.070	0.270
DNAmPAI1	0.250	0.210	0.130
DNAmTIMP1	0.060	0.180	0.230
DNAmPACKYRS	0.080	0.190	0.270
CD8T	0.660	0.980	0.150
CD4T	0.060	0.070	0.140
CD8.naive	0.060	0.410	0.390
CD4.naive	0.460	0.520	0.110
CD8pCD28nCD45RAn	0.130	0.130	0.070
NK	0.110	0.080	0.110
Bcell	0.370	0.630	0.100
Mono	0.070	0.090	0.080
Gran	0.400	0.480	0.120
PlasmaBlast	0.460	0.480	0.120

Table S7. Results of power calculation for 2SR in HPS cohort.

	Power of the study with default effect size		
**Epigenetic Variable**	ALL HPS SAMPLES	ABOVE 60 YEARS	BELOW 60 YEARS
DNAmAge	0.051	0.052	0.050
DNAmAgeHannum	0.052	0.061	0.055
DNAmAgeSkinBloodClock	0.051	0.052	0.050
DNAmPhenoAge	0.053	0.057	0.050
DNAmGrimAge	0.050	0.050	0.052
DNAmTL	0.051	0.051	0.050
DNAmADM	0.054	0.054	0.056
DNAmB2M	0.051	0.053	0.050
DNAmCystatinC	0.051	0.050	0.058
DNAmGDF15	0.054	0.051	0.094
DNAmLeptin	0.053	0.051	0.063
DNAmPAI1	0.054	0.056	0.052
DNAmTIMP1	0.050	0.051	0.052
DNAmPACKYRS	0.050	0.050	0.054
CD8T	0.069	0.104	0.053
CD4T	0.050	0.050	0.051
CD8.naive	0.050	0.052	0.060
CD4.naive	0.053	0.054	0.051
CD8pCD28nCD45RAn	0.051	0.052	0.050
NK	0.051	0.051	0.051
Bcell	0.057	0.072	0.050
Mono	0.050	0.051	0.050
Gran	0.059	0.066	0.051
PlasmaBlast	0.061	0.070	0.052

Table S8. Results of statistical hypothesis testing comparing groups of FM patients and healthy individuals HC, using the 2SR approach. For each variable, mean and median values of residuals in the two groups are reported. Significant p-values are reported in bold.

	**AVERAGE(Age-adj)**			**MEDIAN(Age-adj)**	
**Variable Name**	**FM**	**HC**	**P-value**	**FM**	**HC**
DNAmAge	1.347	0.000	0.595	2.086	2.555
DNAmAgeHannum	-1.569	0.000	0.568	-1.087	1.624
DNAmAgeSkinBloodClock	0.288	0.000	0.910	1.160	3.591
DNAmPhenoAge	0.715	0.000	0.783	0.358	1.344
DNAmGrimAge	-0.125	0.000	0.904	-0.643	0.126
DNAmTL	-0.013	0.000	0.847	-0.008	0.027
DNAmADM	-5.068	0.000	0.175	-7.452	-1.845
DNAmB2M	9172.000	0.000	0.768	9066.000	-393.300
DNAmCystatinC	8788.000	0.000	0.189	7599.000	-3720.000
DNAmGDF15	-11.900	0.000	0.772	-23.550	11.750
DNAmLeptin	355.000	0.000	0.755	610.300	422.600
DNAmPAI1	-3.146	0.000	0.996	78.400	-334.600
DNAmTIMP1	15.120	0.000	0.945	-61.350	136.960
DNAmPACKYRS	-0.175	0.000	0.954	-3.127	-1.860
CD8T	-0.010	0.000	0.365	-0.007	-0.003
CD4T	-0.013	0.000	0.389	-0.025	-0.005
CD8.naive	-9.924	0.000	0.446	-8.611	-7.587
CD4.naive	-66.570	0.000	**0.015**	-54.110	2.098
CD8pCD28nCD45RAn	0.393	0.000	0.682	0.212	0.195
NK	0.019	0.000	0.072	0.014	0.003
Bcell	0.004	0.000	0.521	0.003	0.000
Mono	0.000	0.000	0.961	0.001	0.001
Gran	0.008	0.000	0.728	-0.004	0.010
PlasmaBlast	-0.031	0.000	0.490	-0.007	-0.023

Table S9. Results of power calculation for MLR approach in FM cohort.

**Epigenetic Variable**	**Power of the study with default effect size**
DNAmAge	0.961
DNAmAgeHannum	0.743
DNAmAgeSkinBloodClock	0.913
DNAmPhenoAge	0.865
DNAmGrimAge	0.992
DNAmTL	0.918
DNAmADM	0.910
DNAmB2M	0.982
DNAmCystatinC	0.996
DNAmGDF15	0.934
DNAmLeptin	0.193
DNAmPAI1	0.417
DNAmTIMP1	0.991
DNAmPACKYRS	0.768
CD8T	0.877
CD4T	0.104
CD8.naive	0.904
CD4.naive	0.975
CD8pCD28nCD45RAn	0.566
NK	0.860
Bcell	0.901
Mono	0.242
Gran	0.099
PlasmaBlast	0.731

Table S10. Results of power calculation for 2SR approach in FM cohort.

**Epigenetic Variable**	**Power of the study with default effect size**
DNAmAge	0.555
DNAmAgeHannum	0.191
DNAmAgeSkinBloodClock	0.401
DNAmPhenoAge	0.311
DNAmGrimAge	0.801
DNAmTL	0.417
DNAmADM	0.396
DNAmB2M	0.686
DNAmCystatinC	0.867
DNAmGDF15	0.468
DNAmLeptin	0.054
DNAmPAI1	0.077
DNAmTIMP1	0.778
DNAmPACKYRS	0.206
CD8T	0.322
CD4T	0.051
CD8.naive	0.382
CD4.naive	0.631
CD8pCD28nCD45RAn	0.112
NK	0.291
Bcell	0.366
Mono	0.057
Gran	0.050
PlasmaBlast	0.178

Table S11. Results of statistical hypothesis testing comparing patients with MOH, with EM and healthy individuals HC, using the 2SR approach. For each variable, mean and median values of residuals in the three groups are reported.

	AVERAGE(Age-adj)			MOH vs HC	EM vs HC	MOH vs EM	MEDIAN(Age-adj)		
**Variable Name**	**MOH**	**EM**	**HC**	**P-value**	**P-value**	**P-value**	**MOH**	**EM**	**HC**
DNAmAge	0.132	-2.130	0.000	0.902	0.142	0.108	0.417	-1.327	-0.469
DNAmAgeHannum	-1.510	-2.005	0.000	0.376	0.248	0.787	-0.415	-1.737	-1.007
DNAmAgeSkinBloodClock	0.722	-0.167	0.000	0.505	0.891	0.389	1.119	0.268	0.188
DNAmPhenoAge	0.706	-0.138	0.000	0.633	0.939	0.656	1.318	-1.280	0.077
DNAmGrimAge	-0.659	-1.023	0.000	0.596	0.359	0.766	-2.158	-1.266	-0.986
DNAmTL	0.025	0.006	0.000	0.618	0.911	0.665	0.001	0.011	-0.022
DNAmADM	1.428	4.750	0.000	0.724	0.298	0.399	-0.003	5.114	0.805
DNAmB2M	-34194.517	14069.069	0.000	0.201	0.642	0.094	-48757.402	-8309.935	16770.302
DNAmCystatinC	-5782.656	202.695	0.000	0.333	0.972	0.191	-6108.147	-3745.816	473.814
DNAmGDF15	-10.248	0.124	0.000	0.840	0.998	0.808	-11.840	2.408	62.763
DNAmLeptin	-1757.558	-1987.912	0.000	0.439	0.388	0.875	-2434.097	-2203.351	-530.581
DNAmPAI1	-1366.930	-245.942	0.000	0.194	0.852	0.324	-1358.037	-448.514	899.707
DNAmTIMP1	-49.382	39.536	0.000	0.651	0.725	0.506	-37.597	-14.288	-39.403
DNAmPACKYRS	1.188	-4.106	0.000	0.744	0.197	0.132	-4.129	-5.847	-2.390
CD8T	-0.016	-0.016	0.000	0.104	0.131	0.968	-0.022	-0.013	-0.002
CD4T	-0.005	-0.017	0.000	0.696	0.283	0.407	-0.007	-0.022	0.001
CD8.naive	1.014	-2.841	0.000	0.918	0.781	0.672	6.949	5.835	-11.315
CD4.naive	39.822	-5.026	0.000	0.178	0.867	0.095	40.524	-25.244	-2.077
CD8pCD28nCD45RAn	0.947	1.193	0.000	0.138	0.077	0.709	1.094	0.522	0.478
NK	-0.014	-0.008	0.000	0.215	0.529	0.514	-0.018	-0.014	-0.014
Bcell	0.008	-0.002	0.000	0.165	0.718	0.094	0.005	-0.004	-0.002
Mono	-0.002	0.008	0.000	0.583	0.139	0.070	-0.002	0.006	-0.002
Gran	0.030	0.037	0.000	0.127	0.149	0.741	0.034	0.040	0.008
PlasmaBlast	0.003	0.042	0.000	0.929	0.301	0.391	0.010	0.018	0.015

Table S12. Results of power calculation for MLR approach in MOH/EM cohort.

**Epigenetic Variable**	**Power of the study with default effect size**
DNAmAge	0.970
DNAmAgeHannum	0.959
DNAmAgeSkinBloodClock	0.918
DNAmPhenoAge	0.931
DNAmGrimAge	0.958
DNAmTL	0.885
DNAmADM	0.795
DNAmB2M	0.919
DNAmCystatinC	0.926
DNAmGDF15	0.802
DNAmLeptin	0.923
DNAmPAI1	0.904
DNAmTIMP1	0.913
DNAmPACKYRS	0.901
CD8T	0.687
CD4T	0.818
CD8.naive	0.583
CD4.naive	0.881
CD8pCD28nCD45RAn	0.943
NK	0.806
Bcell	0.904
Mono	0.813
Gran	0.881
PlasmaBlast	0.511

Table S13. Results of power calculation for 2SR approach in MOH/EM cohort.

	**Power of the study with default effect size**		
**Epigenetic Variable**	MOH vs HC	EM vs HC	MOH vs EM
DNAmAge	0.136	0.625	0.395
DNAmAgeHannum	0.246	0.568	0.185
DNAmAgeSkinBloodClock	0.107	0.446	0.267
DNAmPhenoAge	0.124	0.490	0.275
DNAmGrimAge	0.220	0.578	0.219
DNAmTL	0.207	0.364	0.093
DNAmADM	0.140	0.274	0.093
DNAmB2M	0.365	0.370	0.051
DNAmCystatinC	0.294	0.449	0.085
DNAmGDF15	0.136	0.256	0.087
DNAmLeptin	0.281	0.456	0.094
DNAmPAI1	0.421	0.214	0.092
DNAmTIMP1	0.192	0.438	0.144
DNAmPACKYRS	0.051	0.266	0.384
CD8T	0.181	0.091	0.075
CD4T	0.072	0.254	0.175
CD8.naive	0.096	0.120	0.055
CD4.naive	0.322	0.055	0.290
CD8pCD28nCD45RAn	0.298	0.441	0.080
NK	0.279	0.134	0.085
Bcell	0.245	0.059	0.406
Mono	0.141	0.074	0.320
Gran	0.244	0.315	0.061
PlasmaBlast	0.053	0.077	0.113
